# Estimating HIV-1 Fitness Characteristics from Cross-Sectional Genotype Data

**DOI:** 10.1371/journal.pcbi.1003886

**Published:** 2014-11-06

**Authors:** Sathej Gopalakrishnan, Hesam Montazeri, Stephan Menz, Niko Beerenwinkel, Wilhelm Huisinga

**Affiliations:** 1 Institute of Biochemistry and Biology, University of Potsdam, Potsdam, Germany; 2 Graduate Research Training Program PharMetrX: Pharmacometrics & Computational Disease Modelling, Free University of Berlin and University of Potsdam, Berlin/Potsdam, Germany; 3 Department of Biosystems Science and Engineering, ETH Zurich, Basel, Switzerland; 4 SIB Swiss Institute of Bioinformatics, Basel, Switzerland; 5 Institute of Mathematics, University of Potsdam, Potsdam, Germany; University of California San Diego, United States of America

## Abstract

Despite the success of highly active antiretroviral therapy (HAART) in the management of human immunodeficiency virus (HIV)-1 infection, virological failure due to drug resistance development remains a major challenge. Resistant mutants display reduced drug susceptibilities, but in the absence of drug, they generally have a lower fitness than the wild type, owing to a mutation-incurred cost. The interaction between these fitness costs and drug resistance dictates the appearance of mutants and influences viral suppression and therapeutic success. Assessing *in vivo* viral fitness is a challenging task and yet one that has significant clinical relevance. Here, we present a new computational modelling approach for estimating viral fitness that relies on common sparse cross-sectional clinical data by combining statistical approaches to learn drug-specific mutational pathways and resistance factors with viral dynamics models to represent the host-virus interaction and actions of drug mechanistically. We estimate *in vivo* fitness characteristics of mutant genotypes for two antiretroviral drugs, the reverse transcriptase inhibitor zidovudine (ZDV) and the protease inhibitor indinavir (IDV). Well-known features of HIV-1 fitness landscapes are recovered, both in the absence and presence of drugs. We quantify the complex interplay between fitness costs and resistance by computing selective advantages for different mutants. Our approach extends naturally to multiple drugs and we illustrate this by simulating a dual therapy with ZDV and IDV to assess therapy failure. The combined statistical and dynamical modelling approach may help in dissecting the effects of fitness costs and resistance with the ultimate aim of assisting the choice of salvage therapies after treatment failure.

## Author Summary

Mutations conferring drug resistance represent major threats to the therapeutic success of highly active antiretroviral therapy (HAART) against human immunodeficiency virus (HIV)-1 infection. Viral mutants differ in their fitness and assessing viral fitness is a challenging task. In this article, we estimate drug-specific mutational pathways by learning from clinical data using statistical techniques and incorporate these into mathematical models of *in vivo* viral infection dynamics. This approach enables us to estimate mutant fitness characteristics. We illustrate our method by predicting fitness characteristics of mutant genotypes for two different antiretroviral therapies with the drugs zidovudine and indinavir. We recover several established features of mutant fitnesses and quantify fitness characteristics both in the absence and presence of drugs. Our model extends naturally to multiple drugs and we illustrate this by simulating a dual therapy with ZDV and IDV to assess therapy failure. Additionally, our modelling approach relies only on cross-sectional clinical data. We believe that such an approach is a highly valuable tool in assisting the choice of salvage therapies after treatment failure.

## Introduction

The emergence of drug resistant mutants remains a major obstacle to long-term treatment success of highly active antiretroviral therapy (HAART) against HIV-1 [1], [2]. Mathematical models of *in vivo* viral infection dynamics have provided critical insights into HIV-1 disease and therapy by disentangling viral and target cell dynamics [3], [4], quantifying drug class specific effects on viral load decay [5], [6] and elucidating general principles of antiretroviral therapy [7], [8]. Their utility in studying the emergence of drug-specific mutations and resistance, however, is limited by the availability of realistic mutation landscapes. Existing approaches typically use mutation schemes that are unspecific for the drug or coarse-grained [9]–[11]. On the other hand, statistical models of mutational pathways have been used to understand the evolution of drug-resistance *in vivo*, for example, by estimating evolutionary landscapes of viral mutations based on *in vivo* data [12]–[15], establishing genotype–phenotype maps [16] and predicting individual treatment outcomes [17], [18]. These approaches, however, do not integrate details of the viral infection dynamics and the specific actions of different drug classes.

In viral mutational landscapes, the path to resistant mutants that fixate and eventually cause therapy failure typically consists of several intermediate mutants. Understanding the accumulation of mutations and associated genotypic and phenotypic changes is critical for prediction of treatment failure and selection of optimal patient-specific treatments [19]. Additionally, it has been observed that models incorporating quasispecies distributions of HIV-1 mutants can lead to a different qualitative behaviour than what would be expected from simplified mutation models [20].

In a drug-free environment, a viral mutant genotype usually incurs a loss in fitness [21], which is offset by resistance effects in the presence of the drug. This loss in fitness, quantified in terms of a fitness cost, is an important parameter dictating the appearance of mutants and hence affecting viral suppression and therapeutic success [22]. Although fitness landscapes of viruses have been studied for a long time [23]–[25], the paucity and quality of experimental data have always been major limitations [26]. Experimental investigations on viral fitness rely on techniques such as growth competition assays, parallel infection methods, and other replication measurement assays in *in vitro* settings [27]. Replication capacities are typical readouts of such assays and they are considered to be measures of viral fitness [28]. However, there have been controversies over appropriate quantification of viral fitnesses and the clinical relevance of such *in vitro* fitness measures (see [29] for a review). Statistical techniques have been developed and used to estimate relative fitness of viral mutants from longitudinal *in vitro* data [30], [31]. Attempts to estimate fitness parameters in an *in vivo* setting [32], [33] have relied on detailed time course measurements of different mutant strains, which is a severe limitation in the most common situation of sparse data collected during routine clinical diagnostics.

The objective of this article is to enable estimation of *in vivo* fitness parameters from common cross-sectional clinical data by combining and linking statistical methods designed for cross-sectional data with a mechanistic model of virus dynamics, which explicitly accounts for viral fitness. This integration is achieved by (i) learning drug-specific mutational pathways from cross-sectional *in vivo* data and modelling viral infection dynamics on these genotype lattices, and (ii) by coupling the resistance factor, an abundant and accessible *in vitro* measure of drug resistance, to drug efficacy and rate constants of the virus-host dynamics *in vivo*. This approach allows to leverage sparse clinical data for the estimation of *in vivo* fitness characteristics, which is a first step towards analyzing and ultimately predicting clinical outcomes of drug combinations and assessing causes of therapy failure.

Specifically, our statistical approach to estimate mutational pathways from *in vivo* data is based on continuous-time conjunctive Bayesian networks [16]. The viral infection is described based on an established and validated viral dynamics model [6], [34] that explicitly allows for the incorporation of the action of all approved drug classes. Viral resistance is included via drug-specific resistance factors estimated from *in vitro* data by isotonic regression models. The integration is finally achieved by a quantity common to both approaches, the estimated/predicted waiting time for different mutations.

There has been great interest in performing simulations of antiretroviral therapy to assess treatment outcome. Recent studies [35] have shown how even monotherapy simulations using simple viral dynamics models can yield valuable insights on treatment failure and answer clinically relevant questions pertaining to combination therapy. However, implementing multiple-drug therapy with realistic mutational pathways remains a limitation in this regard. Our modelling approach extends naturally to multiple drugs and is a step towards using sparse clinical data effectively to simulate treatment regimens.

We estimate fitness characteristics of mutants for two antiretroviral drugs from two different major drug classes: zidovudine (ZDV), a nucleoside reverse transcriptase inhibitor, and indinavir (IDV), a protease inhibitor. Then, we characterize the interplay between fitness costs and resistances in mutant selection during therapy by computing selective advantages of different mutant genotypes. Finally, we illustrate how our model extends to multiple drug therapy by simulating a dual therapy with ZDV and IDV and examine reasons for virological failure in such a setting.

## Results

### Fitness characteristics of ZDV resistant mutants

We used a dataset obtained from the Stanford HIV Drug Resistance Database described in [36] to estimate possible mutational pathways and phenotypic resistance levels under the selective pressure of ZDV. This dataset consists of 1392 observations of HIV reverse transcriptase (RT) genotypes and associated measurements of phenotypic resistance to ZDV. Phenotypic resistance levels are defined as the logarithm of the fold-change in virus' susceptibility to the drug in comparison to the wild type. We focussed on the key thymidine-analog mutations (TAMs) that arise under ZDV monotherapy: 41L, 67N, 70R, 210W, 215Y and 219Q, where, for instance, 41L denotes the presence of leucine (L) at position 41 of the HIV RT. The genotypes considered were classified into those that exclusively contain mutations from the well-studied TAM-1 (41L, 215Y, 210W) or TAM-2 (67N, 70R, 219Q) pathways [37], [38], and two mixed mutant genotypes that contain mutations from both pathways (with cross-TAM profiles). Mixed mutants are observed to generally occur with a lower frequency [39].

**Figure 1:**
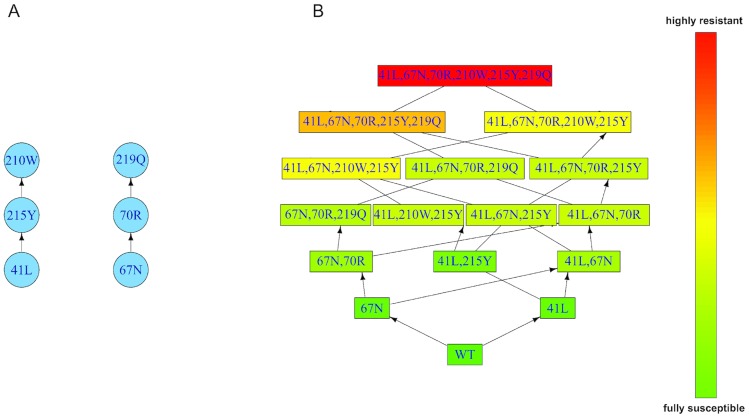
Partially ordered set (poset) and induced genotype lattice for mutations associated with resistance to ZDV. **A**. Poset of resistance development to ZDV. Vertices represent mutations and directed edges represent the order constraints of mutation accumulation. We observed the clustering of thymidine analog mutations (TAMs) along the two classical TAM-1 and TAM-2 pathways that is well-known under ZDV therapy [37], [38]. The left arm of the poset (mutations 41L, 215Y and 210W) is the TAM-1 pathway, while the right arm (mutations 67N, 70R and 219Q) is the TAM-2 pathway. **B**. Genotype lattice of mutants induced by the poset of mutations in **A**. The vertices represent the genotypes that are compatible with the poset in **A**. Predicted levels of phenotypic resistance are color-coded (green, fully susceptible; red, highly resistant). Please see Supplementary Table S2 in Supporting Information for the waiting times of mutations.

Using this dataset, the resistance factors of the genotypes and the partially ordered set (poset) of resistance mutations were estimated using isotonic conjunctive Bayesian network (I-CBN) models. In conjunctive Bayesian networks, a partial order is used to encode dependencies among mutations. A genotype is formally defined as a subset of mutations. The set of genotypes compatible with the order constraints of the poset is called the genotype lattice (Figure 1). In the I-CBN model, isotonic regression is used to associate to each genotype a phenotypic drug resistance level in such a way that resistance levels are non-decreasing along any mutational pathway in the genotype lattice. Next, we used continuous time conjunctive Bayesian networks (CT-CBN) to estimate the rate at which each mutation establishes in the viral population. The fixation times of mutations are assumed to follow independent exponential distributions. The waiting process for a mutation begins only when all its parent mutations in the poset have been established. The data needed for the estimation of this model is a list of genotypes. In the CT-CBN model, genotypes are assumed to be observed after an unknown sampling time. The sampling times are themselves assumed to be random and exponentially distributed. Since we do not know explicitly the time points at which the mutations have occurred, we used the censored CT-CBN model to estimate the fixation rates [40] (See Methods for details).

We used a drug efficacy of 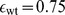 on the wild type (corresponding to a drug concentration of 3 times the IC50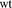) to illustrate our results. This value was chosen to match average nadir values in viral load after ZDV monotherapy (a drop of 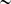
1 log unit from baseline, within 7–10 days of therapy and a nadir at 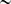
3 weeks) [41].

**Figure 2:**
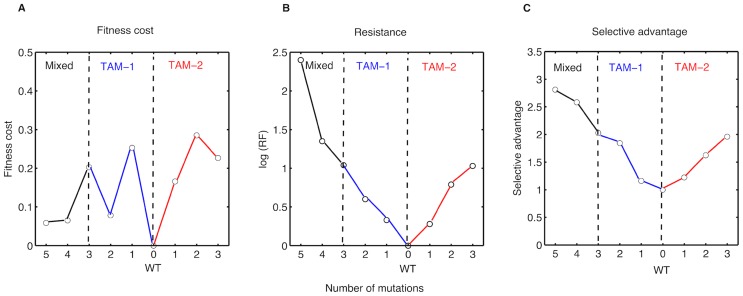
Fitness costs, resistance factors, and selective advantages of mutants arising under ZDV therapy. **A**. Estimated fitness costs (normalized by setting fitness cost of wild type to 0), **B**. Resistance factors (normalized by setting resistance factor of wild type to 1), on a logarithmic scale, and **C**. Estimated selective advantages (normalized by setting selective advantage of wild type to 1) of ZDV mutants. In **A**, **B** and **C**, the x-axis depicts the number of mutations. The TAM-1 mutants (joined by blue solid lines) are to the left and TAM-2 mutants (joined by red solid lines) are to the right of the wild type. The mixed mutants (joined by black solid lines) are to the left of the TAM-1 mutants. The TAM-1, TAM-2 and mixed mutants are separated by vertical dashed lines.

The estimated fitness costs and selective advantages (Table 1 and Figure 2) were in excellent agreement with established knowledge from several *in vitro* assays and some *in vivo* observations. The agreement holds true for general reported ranges of fitness costs (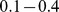) [33], [42]–[44] as well as for statements concerning specific mutations. For example, it is well known that the addition of the 210W mutation into a {41L, 215Y} backbone has opposing effects on fitness depending on the presence or absence of ZDV. In the absence of ZDV, the triple mutant {41L, 210W, 215Y} has been observed to be less fit than {41L, 215Y}, while the introduction of ZDV causes a reversal, i.e., the triple mutant becomes fitter than the double mutant [45], [46]. This was well-reflected in our estimates (Table 1). We observed that this reversal in fitness upon adding ZDV, was because of the higher fitness cost of the triple mutant being more than offset by the resistance acquired in the presence of drug. This can be seen by comparing the selective advantages and fitness costs for the corresponding double and triple mutants in the TAM-1 pathway (Figure 2). TAM-1 mutations are known to occur at almost double the frequency of TAM-2 mutations [47]. This difference was reflected in our results by mutants containing TAM-1 mutations having lower fitness costs than those containing TAM-2 mutations. Additionally, we also observed that the selective advantages of TAM-1 mutants is higher, on average, than their TAM-2 counterparts. This is also in concordance with observations that TAM-2 mutations accumulate only after much longer durations of monotherapy with ZDV [39].

**Table 1:** Estimated fitness costs for ZDV mutants.

Mutant	log RF	Fitness cost	Comment
WT	0	0	Wild type
{67N}	0.28 (0.15, 0.49)	0.17 (0.14, 0.24)	TAM-2
{67N, 70R}	0.79 (0.59, 0.99)	0.29 (0.27, 0.36)	TAM-2
{67N, 70R, 219Q}	1.03 (0.95, 1.11)	0.23 (0.18, 0.30)	TAM-2
{41L}	0.32 (0.18, 0.52)	0.25 (0.17, 0.34)	TAM-1
{41L, 215Y}	0.60 (0.53, 0.65)	0.08 (0.07, 0.11)	TAM-1
{41L, 210W, 215Y}	1.04 (0.95, 1.09)	0.20 (0.16, 0.26)	TAM-1
{41L, 67N, 210W, 215Y}	1.35 (1.30, 1.42)	0.07 (0.05, 0.08)	Mixed
{41L, 67N, 70R, 210W, 215Y, 219Q}	2.40 (1.73, 2.79)	0.06 (0.04, 0.07)	Mixed

Estimated resistance factors (on a logarithmic scale, log RF, column 2) and fitness costs (column 3) of TAM-1 and TAM-2 mutants and two mixed mutants arising during ZDV therapy. In parentheses, are the 95% confidence intervals for the estimates obtained from 200 bootstrap samples (where we resampled with replacement from the list of statistical waiting times and re-estimated fitness costs).

The presence of 41L together with 215Y is a strong predictor of virological failure in patients on ZDV monotherapy [48]. We estimated a low fitness cost for this TAM-1 double mutant and also observed the presence of these two mutations in mutant genotypes contributing to therapy failure.

Our estimated fitness costs were also supported by other *in vitro* investigations on the order of fitness values, such as the TAM-1 triple mutant {41L, 210W, 215Y} being fitter than its TAM-2 counterpart [43] and the TAM-2 double mutant {67N, 70R} being less fit than the single mutant {67N} [49]. Notably, we concurred with the observation in [49] that the occurrence of 70R in a 67N or {67N, 219Q} backbone has a significant cost.

We further studied parameter identifiability by considering an ensemble of fits. All fits with a root mean squared deviation (RMSD) of 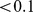 between the normalized statistical and mechanistic waiting times, were treated as equally valid (see Methods). Across all such valid fits, we observed a strong and statistically significant Spearman rank correlation of the estimated fitness costs (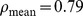, p = 0.020) and selective advantages (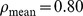, p = 0.017). This indicated that our predictions on the ranking of fitness costs and selective advantages of the different mutant genotypes were strongly conserved. Additionally, in about 90% of the valid fits, we found that the average fitness cost of TAM-1 mutants was less than that of TAM-2 mutants, while in approximately 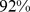 of valid fits, the deleterious effect of 210W inserted in a {41L, 215Y} backbone in the absence of ZDV was preserved. Similarly, we examined the validity of each of our conclusions and found that they were all well-conserved across the valid fits (see Supplementary Table S3 and Supplementary Figure S1 in Supporting Information for details). Moreover, since the parameters of the virus dynamics model are subject to uncertainties, we examined the validity of our predictions under uncertainty of all the viral turn-over parameters, considering perturbation of up to 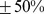. Our model predictions remained robust even under these parameter perturbations (see Supplementary Text S1, Supplementary Figure S2 in Supporting Information for details). Additionally, we also tested the impact of variability of RFs estimated in the first-stage, on the estimation of fitness costs. Again, the order in the estimated fitness costs remained preserved (see Supplementary Text S1 and Supplementary Figure S6).

**Figure 3:**
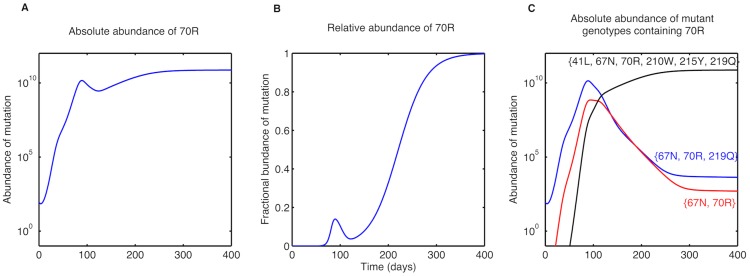
Abundance of the 70R mutation and mutant genotypes with 70R under ZDV therapy. **A**. Absolute abundance (in numbers) of the 70R mutation. **B**. Relative abundance of the 70R mutation in the viral population. The transient appearance and eventual fixation of the mutation 70R can be seen. **C**. Absolute abundance (in numbers) of mutant genotypes containing the mutation 70R. The absolute abundance of a certain mutation is calculated by adding all mutant genotypes containing the mutation.

In addition to estimated fitness characteristics, the viral load time courses predicted by our model gave insights into the dynamics of different mutations. In the TAM-2 pathway, we observed a transient disappearance of mutation 70R before its eventual fixation, as was reported earlier [50]. This phenomenon is attributed to the competition between TAM-1 and TAM-2 mutations: the mutation 70R appears initially and is then outcompeted by 215Y. 70R later fixates in the population after being associated with 67N and other TAM-1 mutations (Figure 3). We also concurred with studies [11] attributing the initial rebound after ZDV monotherapy to insufficient suppression of the wild type, rather than the early selection of mutations.

### Fitness characteristics of IDV resistant mutants

**Figure 4:**
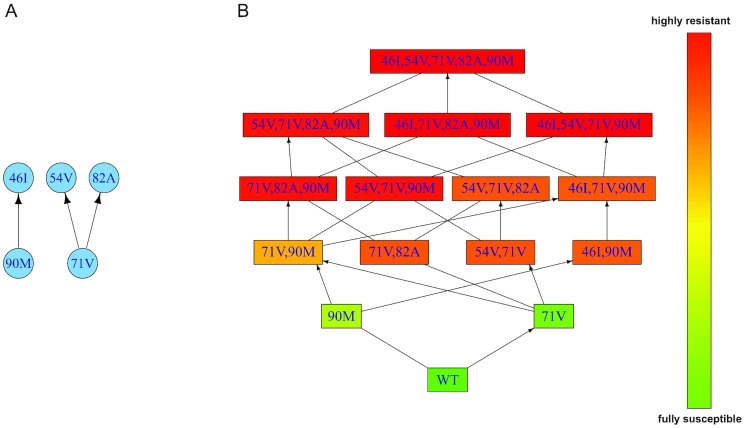
Partially ordered set and induced genotype lattice for mutations associated with resistance to IDV. **A**. Poset of the continuous time conjunctive Bayesian network for resistance development to IDV. **B**. The genotype lattice of mutants induced by the poset in **A**. The vertices represent the genotypes that are compatible with the poset in **A**. The predicted levels of phenotypic resistance are color-coded (green =  fully susceptible, red  =  highly resistant). Please see Supplementary Table S2 in Supporting Information for the waiting times of mutations.

We again used the Stanford HIV Drug Resistance Database [36] to estimate the poset (Figure 4A) and genotype lattice (Figure 4B) of mutations associated with resistance to indinavir (IDV), a protease inhibitor, and corresponding resistance factors of IDV mutants. As for ZDV, the poset, the genotype lattice, and the rate of fixation for each mutation were determined by the I-CBN and CT-CBN models. The dataset for IDV consists of 2170 observations of HIV reverse transcriptase (RT) genotypes and their paired measurements of phenotypic resistance to IDV. We focussed on the five mutations 46I, 54V, 71V, 82A, and 90M. Four of these (46I, 54V, 82A and 90M) are among the most frequent primary (major) mutations reported in the Stanford HIV Drug Resistance Database under IDV therapy [36]. We chose 71V to represent a common secondary (minor) mutation to study possible compensatory fitness effects. We used a drug efficacy 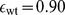 on the wild type to illustrate our results. This value was chosen to match average nadir values in viral load after IDV monotherapy (a drop of 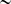
1–1.5 log units within 3–4 weeks of therapy) [51], [52].

**Figure 5:**
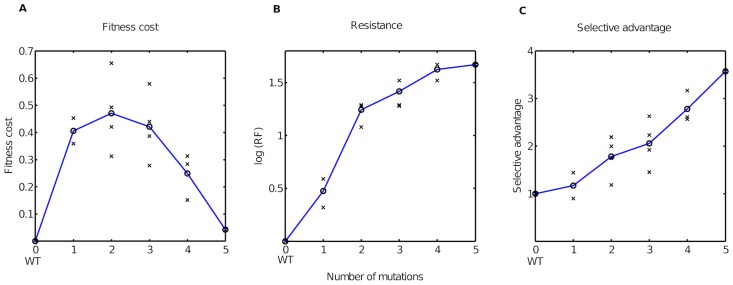
Fitness costs, resistance factors and selective advantages of mutants arising under IDV therapy. **A**. Estimated fitness costs (normalized by setting fitness cost of wild type to 0), **B**. Resistance factors, on a logarithmic scale (normalized by setting resistance factor of wild type to 1), and **C**. Estimated selective advantages (normalized by setting selective advantage of wild type to 1) of IDV mutants. In **A**, **B** and **C**, the x-axis depicts the number of mutations. Black crosses represent the values for the different mutant genotypes, while the blue solid line represents the average of fitness costs, resistance factors and selective advantages across all mutant genotypes with a given number of mutations.

The estimated fitness costs, resistance factors and selective advantages (Table 2 and Figure 5) agreed well with reported experimental findings. In general, we observed that early mutations have a high fitness cost, while the accumulation of further mutations succeeded in compensating almost entirely for this loss in fitness (Figure 5A). This is in agreement with clinical observations that mutations selected early during therapy with protease inhibitors cause impaired protease function and that subsequent accumulation of mutations compensates for this fitness cost [27], [53]. A striking behaviour that we observed was the presence of staircases in the fitness landscape, which has also been described earlier [54]. We observed a monotonic increase of the average selective advantages of the mutants with increasing number of mutations (Figure 5C). This observation provides additional reasoning for the accumulation of mutations during IDV therapy. Notably, the high fitness costs for the double and triple mutants (Figure 5A) were not sufficient to deter their occurrence, as the fitness costs were well-offset by resistance (Figure 5B), which facilitated further climbing of the fitness landscape by accumulating mutations (Figure 5C).

**Table 2:** Estimated fitness costs for IDV mutants.

Mutant	log RF	Fitness cost, 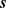 (95% CI)	Type
WT	0	0	Wild type
{90M}	0.59 (0.56, 0.72)	0.36 (0.33, 0.38)	Single point (M)
{71V}	0.32 (0.26, 0.48)	0.45 (0.38, 0.50)	Single point (m)
{46I, 90M}	1.28 (1.26, 1.37)	0.66 (0.56, 0.70)	Double (MM)
{71V, 90M}	1.08 (1.04, 1.18)	0.31 (0.29, 0.34)	Double (mM)
{54V, 71V}	1.29 (1.26, 1.37)	0.49 (0.47, 0.54)	Double (Mm)
{71V, 82A}	1.29 (1.26, 1.37)	0.42 (0.39, 0.44)	Double (mM)
{54V, 71V, 82A}	1.29 (1.26, 1.34)	0.58 (0.53, 0.71)	Triple (MmM)
{54V, 71V, 90M}	1.52(1.35, 1.64)	0.28 (0.26, 0.34)	Triple (MmM)
{71V, 82A, 90M}	1.52 (1.45, 1.64)	0.39 (0.32, 0.42)	Triple (mMM)
{46I, 71V, 90M}	1.28 (1.26, 1.34)	0.44 (0.41, 0.47)	Triple (MmM)
{54V, 71V, 82A, 90M}	1.52 (1.45, 1.64)	0.28 (0.22, 0.32)	Quadruple (MmMM)
{46I, 71V, 82A, 90M}	1.67 (1.57, 1.74)	0.31 (0.28, 0.33)	Quadruple (MmMM)
{46I, 54V, 71V, 90M}	1.67 (1.65, 1.74)	0.15 (0.12, 0.17)	Quadruple (MMmM)
{46I, 54V, 71V, 82A, 90M}	1.67 (1.65, 1.74)	0.04 (0.03, 0.05)	Quintuple (MMmMM)

Estimated resistance factors (on a logarithmic scale, log RF, column 2) and fitness costs (column 3) of mutants arising during IDV therapy. In parentheses, are the 95% confidence intervals for the estimates obtained from 200 bootstrap samples (where we resampled with replacement from the list of statistical waiting times and re-estimated fitness costs). Mutant types (column 4) are encoded by one ‘M’ for each major mutation and one ‘m’ for each minor mutation in the genotype.

In addition to these general fitness trends, specific characteristics of particular mutations were also in line with prior findings. The minor mutation 71V is known to play a compensatory role [55]. In our estimates, this was observed by a partial recovery in fitness of the triple mutant {46I, 71V, 90M} compared to the double mutant {46I, 90M} from 0.66 to 0.44 (Table 2). In the presence of IDV, the addition of 54V to a {71V, 82A} backbone is known to not confer a significant advantage [56], and this was reflected by a ratio of approximately 1.3 for the selective advantages of this pair of mutants. Furthermore, as in [57], we noted a higher fitness cost for the single mutant 71V as compared to 90M.

Nijhuis et al. [55] observed the persistence of protease resistant mutants for long periods of time even after the cessation of therapy. They argued that the reversal of the underlying mutations might not be feasible due to lower replication capacities of intermediate mutants upon reversion. Our results support this hypothesis by showing that the most resistant strain that develops after therapy failure is very unlikely to reverse back in the mutational landscape, owing to a fitness barrier encountered in its reversion to the wild type (Figure 5A).

As with ZDV, we studied parameter identifiability by considering an ensemble of fits with an RMSD 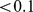 between the statistical and mechanistic waiting times. There was a statistically significant Spearman rank correlation (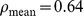, p = 0.014) between the best estimate of fitnesses and all other valid fits. We found the average fitness estimates (Figure 5C) to be very strongly conserved (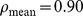, 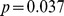). We also examined each of the results above and observed consistency across the valid fits (see Supplementary Table S3 and Supplementary Figure S1 in Supporting Information for details). For example, in 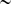
77% of the valid fits, 71V was observed to play a compensatory role by lowering fitness costs, while in 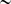
65% of fits, the single mutant 90M was fitter than 71V.

### Dual therapy with ZDV and IDV

There is great interest in using viral dynamics models to study antiretroviral treatment to assess therapy outcomes and simulate clinical trials [35]. Our model extends naturally to multiple-drug therapy. To illustrate this, we performed simulations of a dual antiretroviral therapy with zidovudine (ZDV) and indinavir (IDV). We used the posets of mutations for ZDV and IDV that we have estimated earlier (Figure 1A and Figure 4A, respectively), together with the resistance factors and fitness costs of the different mutant genotypes (see Methods for details). Our goal was to assess treatment outcomes and reasons for failure of the dual regimen. To this end, we used a range of 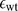 values for ZDV and IDV to account for differential drug effects and adherence patterns, and studied the treatment outcome by monitoring the total viral load. Our simulations enabled us to predict the dominant mutant genotypes at the point of failure (we defined failure at the point when the viral load crossed a threshold of 500 copies/ml). Based on this, we classified failure as being due to wild type, mutations resistant to ZDV, mutations resistant to IDV or mutations resistant to both drugs used.

**Figure 6:**
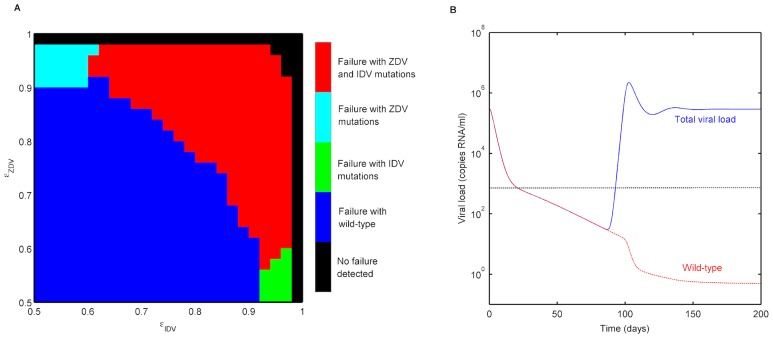
Treatment outcome with ZDV+IDV dual therapy. **A**. Genotypic reasons of treatment failure were assessed in terms of mutations present at point of virological failure. For different combinations of drug efficacies 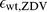 and 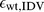, the different genotypic reasons of failure are shown in different colours. The treatment outcome could be a) failure with mutations resistant to both ZDV and IDV, (b) failure with mutations resistant only to ZDV, (c) failure with mutations resistant only to IDV, (d) failure with wild type, and (e) no detection of failure. **B**. Viral load (in copies RNA/ml) under ZDV+IDV therapy with 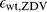 = 0.75 and 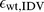 = 0.90. The blue line shows the total viral load, while the red dashed line depicts the wild type. The horizontal black dashed line represents the detection threshold used (500 copies/ml).

We observed that there are different regimes of the individual drug efficacies (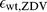 and 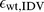) that result in varying causes of failure (Figure 6A). With 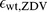 = 0.75 and 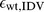 = 0.90, for example, we observed virological failure after 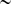
3 months (Figure 6B) due to mutations resistant to both drugs. In this case, the wild type is sufficiently suppressed and declines during the treatment period. However, we identified regions in the 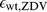-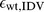 plane, where treatment failure occurred due to insufficient suppression of the wild type. We classified the treatment as having failed owing to the wild type, if the wild type was the dominant genotype at the point of virological failure. We note that the wild type would eventually be out-competed by resistant mutants in all situations. Such situations of failure with the wild type could indicate insufficient drug pharmacokinetics, a low drug efficacy or poor adherence. This would have implications in designing a salvage therapy regimen, subsequent to failure. Additionally, we also observed that there are combinations of (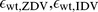), for which failure occurs due to mutations to one of the two drugs (Figure 6A). Our model, thus, enabled prediction of viral evolution under a multiple-drug treatment scenario.

We noted that for a certain 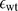 of one drug, predicting the treatment outcome predicted from monotherapy simulations might lead to qualitatively different results, as opposed to using a model with multi-drug therapy. For example, the value of 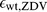 below which failure with wild type is detected was different in ZDV monotherapy simulations. This reiterates the value of implementing multi-drug treatment regimens in *in silico* simulations.

We further emphasize that in order to simulate a certain combination, our approach needs only clinical data from treatment regimens in which the individual drugs are a part. For instance, in the current example of dual therapy with ZDV and IDV, the estimation of resistance factors and fitness characteristics relied on sparse cross-sectional clinical data from treatment regimens that included ZDV and/or IDV (not necessarily both). Subsequent to this, we were able to simulate the dual therapy and assess genotypic reasons of therapy failure.

## Discussion

We have presented an HIV-1 infection dynamics model with statistically learned drug-specific *in vivo* mutational landscapes. Our approach relies on typical and frequently available clinical data, which consists of left-censored observations, as opposed to extensive time course measurements of different mutant genotypes, that are generally scarce. Mutations are detected through their appearance in specific mutant genotypes and our model describes how their dynamics is coupled. Although there is considerable debate on appropriate measures of fitness and clinically relevant quantifiers [29], evidence exists for the influence of fitness on transmission efficiency [58], plasma viral load [59] and treatment interruption outcomes [60]. Assessing fitness characteristics has also been shown to have *in vivo* clinical relevance as correlations between fitness measures and standard treatment outcome markers, such as viral load, [61] have been demonstrated. We estimated fitness characteristics of drug resistant HIV-1 mutants and illustrated our results for ZDV, a nucleoside reverse transcriptase inhibitor, and IDV, a protease inhibitor. Our estimated fitness costs and selective advantages showed excellent agreement with experimental knowledge.

So far, mechanistic modelling of HIV infection has mainly used fixed fitness costs with the infection dynamics being examined for different values of these fitness costs [6], [9]. However, it is well known that fitness landscapes of HIV-1 are highly rugged [26]. Earlier work characterizing *in vivo* fitness characteristics has relied on detailed viral load measurements of different mutant strains, which is rarely possible in realistic clinical situations. Our approach utilized statistical learning methods to estimate mutational landscapes from clinical data that were then incorporated into a mechanistic viral dynamics model. Our results are also in agreement with an earlier study [11] where a mechanistic modelling approach was used to fit drug efficacy and fitness parameters to clinically observed mutant data under zidovudine and lamivudine therapy. As observed in this study, we also noted that the lack of adequate suppression of the wild type strain contributes significantly to the initial rebound in the viral load. In this scenario, the wild type strain initially rebounds leading to virological failure and then later declines after being out-competed by the mutants. In comparison to [11], our model is a more detailed version in the mechanistic sense and further accounts for more realistic mutation pathways. For the protease inhibitor IDV, our results clearly demonstrated the incentive for the accumulation of mutations by HIV-1 in spite of significant losses in fitness incurred by the first few mutations. Interestingly, the high fitness costs of double and triple mutants do not deter their occurrence, and their appearance paves the way for later mutants with higher fitness [53]. In line with earlier studies [55], we observed a fitness barrier that prevents reversion to wild type upon cessation of therapy.

The simulation of antiretroviral treatments has generated interest, particularly in the context of assessing therapy failure and in explaining puzzling clinical observations. For example, clinical trials have shown [62], [63] that some protease-inhibitor containing regimens fail without mutations being detected in the protease region of the HIV genome. Bloomenfeld et al. [35] used a simple viral dynamics model and information on mutations and drug pharmacokinetics from literature to simulate monotherapies and deduced that the short time spent by protease-resistant mutants in the mutant-selection window was responsible for lack of selection of mutations in the protease regimens. However, the inclusion of only single point mutants is a limitation in modelling long-term treatment and in ascertaining the impact of a certain failed regimen on potential salvage therapies. Our model includes different target cells (T-cells and macrophages), a latent reservoir, multiple major drug-resistance mutations and extends to combination therapies, and hence represents a first step in using viral dynamics models informed by mutations and resistance through statistical learning from clinical data, to assess and understand the impact of a failed regimen. The long-lived infected macrophages and latently infected cells in the virus dynamics model contribute to different later stages of viral decay and their impact would be significant in the analysis of multiple-drug regimens.

The presented viral infection dynamics model incorporating drug-specific *in vivo* mutation landscapes aimed at capturing the complex competition dynamics between the different mutant strains. It was based on a simplified representation of drug pharmacokinetics (PK) and effect. If detailed data on drug PK and patient-specific viral load dynamics and baseline characteristics are available, a population-pharmacokinetic/pharmacodynamic analysis would be the appropriate approach to account for inter-individual variations [64]. In the absence of such detailed data, we assessed the impact of time-varying drug concentrations on our model predictions by integrating a simple two-compartment PK model of ZDV. The mechanistic predicted waiting times retained a high and significant correlation with the average statistical waiting times (details in Supplementary Text S1, Supplementary Table S4 and Supplementary Figure S3). Hence, our simplifying assumption of a constant drug concentration and effect seems reasonable and is in line with most prior analyses [65]. Further, while deterministic simulations represent the average dynamical behaviour of the system, stochastic effects need to be incorporated using numerical hybrid algorithms to explain the variability in clinical data. We performed an initial analysis by using such a hybrid deterministic-stochastic algorithm [66], that switches from deterministic to stochastic regime below a certain threshold (separately for each reaction). While, we observed a delay in the appearance of certain mutations in agreement with previous observations [67], the model predictions remained robust with regard to the order of appearance of mutations and the predicted waiting times continued to be significantly correlated with the statistical waiting times used to fit the model (details in Supplementary Text S1, Supplementary Table S6 and Supplementary Figure S5).

There are several mechanisms of resistance in HIV-1 infection. In addition to the mechanisms included in the two-stage virus dynamics model, features such as the compensatory Gag mutations [68] and other compensatory mechanisms adopted by HIV-1 have also been described, including frame-shifts in the Gag region that increase viral protease expression levels [69]. These effects can be integrated by including information on the Gag region into the mutational scheme and this extended model may then partially account for higher observed fitness levels of some mutants.

In summary, we have presented a new approach to model HIV-1 infection dynamics that incorporates drug-specific *in vivo* mutational landscapes and allows for the estimation of mutant fitness characteristics. Importantly, it relies only on cross-sectional clinical data and, as demonstrated, extends naturally to combination therapies. We believe that it is a promising approach to analyze treatment outcomes with drug combinations or to study optimal switching strategies.

## Methods

### Mechanistic viral dynamics model

**Figure 7:**
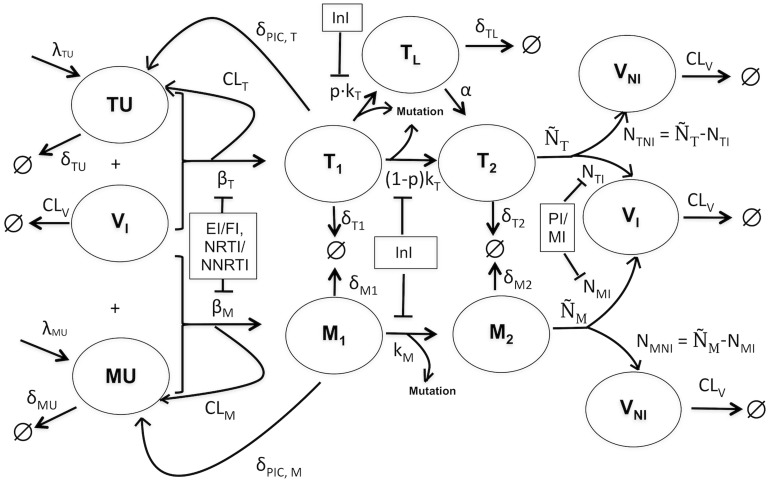
Two stage mechanistic model of *in vivo* HIV-1 infection dynamics [6]. Target cells TU (T-cells) and MU (macrophages) can be infected by infective viruses 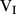 (with effective infection rate constants 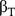 and 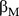), resulting in early stage infected cells 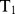 and 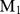, respectively. Infection can also be unsuccessful after fusion of the virus, rendering the cell uninfected and thereby eliminating the virus (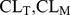). 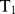 and 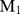 can also possibly return to uninfected states by destruction of essential viral proteins or DNA prior to integration (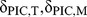). 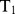 cells can enter into a latent state 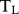 (with probability 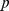) that can get re-activated with a rate constant 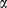. Integration of viral DNA in the host genome proceeds with reaction rate constant 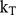 in the T-cells and 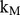 in the macrophages, resulting in late stage infected T-cells 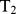 and macrophages 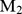, respectively. The infected 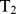 cells release new viruses (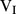) and non-infective (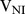) viruses (with rate constants 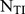 and 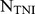, respectively) while the infected 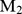 cells release new infective and non-infective viruses (with rate constants 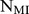 and 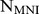, respectively). Target cells TU and MU are produced by the immune system at constant rate with rate constants 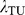 and 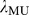, respectively. 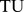, 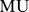, 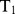, 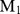, 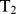 and 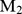 can be cleared by the immune system with reaction rate constants 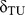, 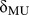, 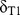, 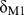, 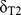 and 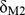, respectively. Viruses are cleared by the immune system with a rate constant 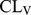. Mutations are modelled to occur at the stage of integration of the viral DNA. The incorporation of the various drug classes is indicated by the inhibition of corresponding processes: EI/FI - entry/fusion inhibitors, NRTI/NNRTI - nucleoside/non-nucleoside reverse transcriptase inhibitors, InI - integrase inhibitors, PI/MI - protease/maturation inhibitors.

The viral infection cycle was described by the two-stage model presented in [34]; see Figure 7 for a graphical representation and description, Supplementary Text S1 for the corresponding system of ordinary differential equations (ODEs) and Supplementary Table S1 for the parameters used. The model allows for integrating drug-specific mutation schemes and the actions of all approved antiretroviral drug classes including reverse transcriptase inhibitors (RTIs), protease inhibitors (PIs) and integrase inhibitors (InIs).

Mutations in the viral genome occur during the process of reverse transcription, but manifest themselves only after the viral DNA has been integrated into the host genome. Hence, mutations were modelled to occur between early infected cells (first stage 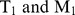) and late infected cells (second stage 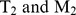). We considered only mutation events between genotypes differing by a single amino acid site, owing to the fact that, though multiple amino acid changes are simultaneously possible, the probability of such events is very low. The probability 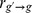 of a mutation that changes the genotype from 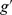 to 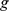 was assumed to be 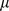 for a single underlying base-pair mutation and 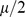 for a double underlying base-pair mutation, where 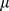 denoted the probability of mutation per base-pair per cycle of replication (see Supplementary Text S1 for details, in particular regarding the choice of 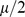). Since mutations are primarily a result of error-prone reverse transcription [70], forward and backward mutations were considered. The average error rate in viral reverse transcription is about 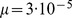 mutations per nucleotide per cycle of replication; and two-thirds of these mutations are known to be base-pair substitutions [70]. In agreement with [11], we used the following nucleotide-specific mutation rates: for a G 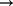 A nucleotide change, we set a mutation rate of 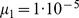 (because about half of the base-pair mutations are of this type [70]), for mutations involving nucleotide changes A 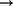 G, we used a lower rate of 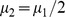, and for transversion and other mutations (involving a change from a purine to a pyrimidine or *vice-versa*), we set 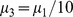.

As with infectious viruses, we also included different mutant strains of non-infectious viruses, since viral detection assays do not distinguish between infectious and non-infectious viral particles.

The effect of an antiretroviral drug on a viral genotype 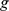 was modelled by a fractional reduction of the targeted process, characterized by the drug efficacy parameter
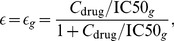
(1)where 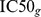 denotes the drug concentration at which the fractional reduction is 50%. The subscript 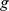 indicates that the 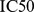-value and thus the drug efficacy was assumed to be genotype dependent (see below). Denoting by 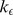 and 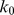 the rate constants of the targeted process, in the presence and absence of the drug, respectively, drug action was modelled by
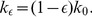
(2)The two-stage model in [34] was derived from a more detailed viral infection model by model reduction (see [6] for details). As a consequence, the processes of infection of T-cells and macrophages (with rate constants 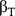 and 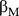, respectively) and production of new infectious and non-infectious viruses from infected T-cells (with rate constants 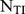 and 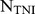, respectively) and from infected macrophages (with rate constants 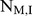 and 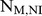, respectively) are lumped processes, integrating several subprocesses. For example, the infection process comprises the subprocesses of receptor binding, fusion and reverse transcription. The consequences of model reduction have to be taken into account when modelling the actions of drugs targeting some of these subprocesses in the two-stage model, resulting in an additional model reduction factor 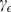. This is analogous to the model of competitive inhibition in the context of the Michaelis-Menten approximation of substrate conversion by an enzyme: Defining the unperturbed rate constant as 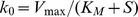, the model of competitive inhibition stated in terms of the inhibitory concentration 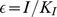 in units of 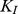 would be: 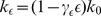 with 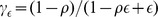 and 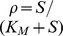. For the drug classes of our interest, namely RTIs and PIs, eq. (2) becomes
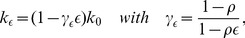
(3)where 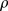 denotes the probability of successful reverse transcription (for RTIs) or the probability of successful viral maturation (for PIs); and 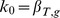 and 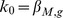 for RTIs, and 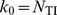 and 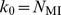 for PIs. For further details, we refer to Supplementary Text of [6].

The advantage of decreased drug-susceptibility of a mutant genotype 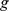 is typically counter-balanced by a reduction in the fitness of the viral strain [21]. This was quantified in terms of the fitness costs 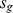. We made the common assumption that a mutation in a part of the genome that is associated with a certain process of the viral replication cycle caused a drop in the rate of only this process. For example, we assumed that a mutation in the reverse transcriptase part of the HIV-1 genome resulting in a mutant genotype 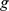 lowers only the rate of reverse transcription. This is a reasonable assumption at least for HIV-1 [71], as most resistance mutations occur in the region of the genome that is coding for the drug target. Hence, any cost due to a resistance mutation is also most likely to be incurred on the function of this region.

Aiming at the integration of *in vitro* measurements of drug- and strain-specific resistance factors, we parametrized all mutant genotypes with reference to the wild type. This also allowed for the estimation of genotype-specific fitness costs 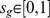. We denoted by 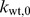 the rate constant of the targeted process in the wild type in the absence of any drug. For a genotype 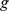 and drug efficacy 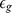, the rate constant of the targeted process was defined as
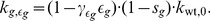
(4)The first factor accounts for the reduction in activity due to drug action. The genotype-dependent drug efficacy 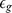 is given by eq. (1), where
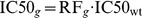
(5)was defined in terms of the wild type 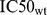-value and the resistance factor 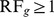 accounting for the increase in drug-resistance. The second factor in eq. (4) accounts for the reduction in activity due to loss of fitness of the mutant genotype.

In the absence of drug (i.e., 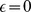), resistance plays no role and it is clear from eq. (4) that the overall rate of infection for the mutant is lower than that of the wild type, such that the wild type eventually outcompetes the mutant. In the presence of drug, the fitness of a mutant genotype 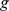 depends on the dynamic interplay between the two factors: the fitness cost 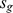 and the resistance factor 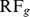. We quantified the overall fitness of a mutant relative to the wild type based on the selective advantage (similar to [72]) as
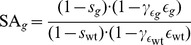
(6)with 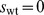 by definition. If 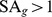, the mutant 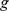 has a replicative advantage over the wild type in the presence of drug.

### Statistical model of mutation accumulation

The structure of the mutational landscape determined the scheme of mutations that entered the system of ODEs in the mechanistic model (see Supplementary Text S1). We estimated the mutational scheme from clinical data based on a continuous-time conjunctive Bayesian network (CT-CBN) [16]. The CT-CBN is defined by a partially ordered set (poset) of mutations, denoted by 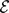 with order relation 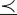 and by the rate of accumulation (i.e., generation and fixation) of each mutation. The poset specifies the order in which mutations can accumulate in the viral population. The relation 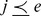 indicates that mutation 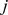 has to occur before mutation 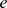. In this case, we call 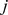 a parent of 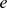 and denote the set of all parents of 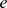 by 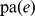. A subset 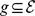 of mutations is called a genotype. The genotype lattice, denoted by 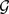, consists of all genotypes that are compatible with the order constraints of 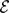. It defines the subset of possible mutational pathways (see Figure 1 for illustration).

We assume that the occurrence times of mutations follow independent exponential distributions and that the exponential waiting process for a mutation starts only after occurrence of all of its parent mutations in the poset. Formally, for each mutation 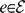, we define a random variable 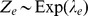. Then we define the statistical waiting times 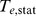 recursively as the random variables
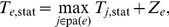
(7)for each mutation 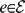. In practice, the times of occurrence of mutations are rarely observed and we can solely measure which mutations have been observed at a particular time point, called sampling time or genotyping time. The HIV genome can only be determined if the total viral load is greater than a detection limit 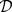 = 500 copies/ml of viral RNA [73]. Hence, in HIV drug resistance development, genotyping can only be performed after therapy failure which is defined as the viral load increasing above the detection limit of a genotypic assay (often 50 copies per mL), thereby precluding the observation of the occurrence of each individual mutation. Sampling times are assumed to be themselves random. In the CT-CBN model, the sampling time 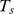 is assumed to be exponentially distributed, 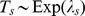, and independent of the poset. It is noteworthy that waiting time for the sampling event starts from the onset of therapy (time zero). However, these time points are not available in the Stanford HIV Drug Resistance Database. In this setting, if we observe genotype 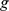, then 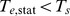 for all 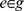 and 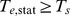 for all 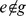.

We assign a resistance factor 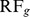 to each genotype 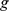. Resistance factors are typically measured experimentally *in vitro* by fluorescence-based replication assays [74]. In practice, they are available for some genotypes, but not necessarily for all genotypes of interest. In general, resistance factors observed for a genotype defined by a subset of mutations will vary due to experimental noise and different genetic backgrounds. Supplementary Figure S5 shows the variability of measured resistance factors under ZDV and IDV therapies. To derive a general model, we employ isotonic-CBNs (I-CBNs) and learn a mapping from the genotype space to the drug resistance phenotype space [16]. I-CBN models assume that resistance factors are non-decreasing over the genotype lattice in the direction of evolution. Because of the monotonicity assumption, the regression problem is constrained, which serves as a means of regularization.

The estimation of the statistical models was performed in two steps. In the first step, I-CBN models were estimated separately using 1392 and 2170 *in vitro* cross-sectional genotype–phenotype observations from the Stanford HIV Drug Resistance Database [36] for ZDV and IDV, respectively. The genotype–phenotype observations were restricted to the 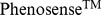 [75] or the 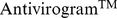 [76] assays. For ZDV, the I-CBN was applied to mutations 41L, 67N, 70R, 210W, 215Y, and 219Q in the reverse transcriptase region of the viral genome. For IDV, protease mutations 46I, 54V, 71V, 82A, and 90M were analyzed. Each I-CBN model included a poset of mutations and the estimated resistance factors. In the second step, based on the estimated poset, the rate parameters 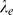 of the CT-CBN models were estimated from the cross-sectional genotype observations of the Stanford HIV Drug Resistance Database using an Expectation-Maximization algorithm [40].

### Waiting times, parameter estimation and identifiability

Having specified the parameters of the two-stage dynamical model of viral infection (see Supplementary Table S1 and [6]), a mutation landscape of genotypes, resistance factors and drug efficacies, the only unknown parameters of the model are the fitness costs of the different genotypes. These unknown parameters were estimated by comparison of predicted mechanistic waiting times (based on the mechanistic model of viral infection dynamics) to the statistical waiting times eq. (7). We linked the statistical point of view in terms of mutations and the mechanistic point of view in terms of mutant genotypes as follows.

A given mutant genotype 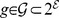 is defined as the set of all mutations 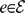 that are manifested in 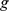. We defined the mechanistic waiting time 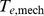 for each mutation 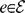, as the earliest time, at which the following two criteria were satisfied: (i) all the mutant genotypes containing the mutation 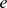 together constituted at least 20% of the total viral population 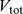; the 20% detection threshold reflected the limitations of the genotyping assay [77]; and (ii) the total viral load was greater than a typical detection limit 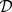 = 500 copies/ml of viral RNA, that enables genotyping [73]. This resulted in the following definition of the mechanistic waiting time of mutation 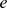:
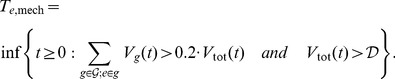
(8)As discussed in the previous subsection, the average waiting times computed from the statistical model 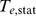 are dependent on the sampling rate 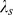, to which we typically did not have access. As a solution to this problem, we compared only the relative time scales of appearance of the different mutations. This was done by normalizing the statistical waiting times by the time to the fastest occurring mutation, and then comparing these to correspondingly normalized mechanistic waiting times.

For simulations of the mechanistic viral dynamics model under therapy, we first performed a pre-treatment steady state computation to estimate levels of different mutants at the onset of therapy. All model simulations were performed with MATLAB R2010b. For estimation of fitness costs, we used the MATLAB optimization function *fminsearchbnd* that is based on the Nelder-Mead simplex direct search algorithm [78] to perform the constrained least-squares estimation
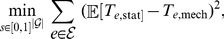
(9)where 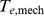 depends on the fitness costs 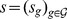 via the system of ODEs specifying the viral dynamics. Fitness costs 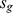 with 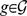 were then defined as the solution of eq. (9). Note that in addition to the constraints on fitness costs, the mechanistic waiting times were also subject to the order constraints imposed by the structure of the mutation poset.

In detailed mechanistic models with nonlinearities, parameter identifiability and sloppiness in estimation is a common concern [79], [80]. To study the relevance of this phenomenon in our setting, we performed 500 repeated estimations by choosing different random initial estimates. We also subsequently performed simulated annealing with the function *simulannealbnd* in MATLAB to test for convergence of our estimates. We did not explicitly observe non-identifiability (indicated by a flat cost function surface in multiple dimensions near the best estimate). However, as in [81], we noted that several rounds of simulated annealing converged to different estimates indicating a rugged search landscape and a possible lack of a well-defined minimum. Following the approach in [81], we considered all fits with an RMSD of less than 0.1 between the mechanistic and statistical waiting times as equally valid (since we estimate the average error in the normalized statistical waiting times data to be 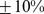) and discussed our results based on this ensemble of fits.

More details on the RMSD threshold and the number of fits considered are provided in the Supplementary Text S1.

### Model simulations for dual therapy

For dual therapy with ZDV and IDV, the poset was constructed by combining the posets of the individual drugs. If 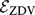 and 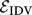 denote the posets under ZDV and IDV monotherapy, then the poset 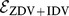 under dual therapy with ZDV and IDV can be written as the disjoint union of the individual posets. That is,
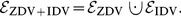
(10)with the partial order relation in 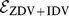 just being the disjoint union of the partial orders in the individual posets.

If 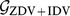 is the genotype lattice induced by the poset 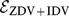 under dual therapy, then a mutant genotype 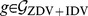 has contributions from two sources towards its fitness cost 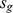 and resistance factor 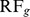
— one from the underlying RT mutations and another due to protease mutations. As before, this simply manifests as changes in reaction rates of appropriate target steps in our viral dynamics model, the only difference being that there are two target steps, instead of one in monotherapy. We assumed mutations in these two regions to be free from fitness and resistance epistasis. This is a reasonable assumption in view of studies indicating that intragenic epistatic effects are more significant than intergenic epistatic effects [28]. We note that if two drugs from the same drug class are to be considered, this either requires some additional assumptions on the extent of epistatic effects or would involve estimation of additional parameters.

For simulations of the dual therapy with ZDV and IDV, we used the variable order ODE-solver *ode15s* in MATLAB R2010b with relative and absolute tolerances of 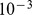, and a non-negativity constraint. To study the effects of changing the drug-efficacy parameters 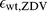 and 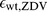, we performed simulations of the dual therapy for 300 days. To classify therapy outcomes, we monitored total viral load and detected failure when the viral RNA exceed 500 copies/ml. Again, we chose this threshold to correspond with genotyping assay limits. We reported detection of no failure if the viral load did not exceed this threshold within our simulation time.

## Supporting Information

Figure S1 **Cumulative histograms of correlations of fitnesses between valid fits and best fit.**
(PDF)

Figure S2 **Distribution of mechanistic waiting times from 1000 simulations of the virus dynamics model.**
(PDF)

Figure S3 **Two compartment pharmacokinetic model for ZDV and fluctuating drug-effect.**
(PDF)

Figure S4 **Variability of resistance factors under ZDV and IDV therapy.**
(PDF)

Figure S5 **Predicted mutational abundance from 500 hybrid deterministic-stochastic simulations of IDV monotherapy.**
(PDF)

Figure S6 **Cumulative histograms of correlations of fitness costs between fits with varying RFs and the best fit.**
(PDF)

Table S1 **Parameters of viral dynamics model used in the simulations.**
(PDF)

Table S2 **Statistical and mechanistic waiting times to observe mutations under ZDV and IDV monotherapy.**
(PDF)

Table S3 **Conservation of estimated fitness characteristics of ZDV and IDV mutants amongst valid fits.**
(PDF)

Table S4 **Statistical and mechanistic waiting times to observe mutations under ZDV monotherapy with ZDV concentration described by a two-compartment pharmacokinetic model.**
(PDF)

Table S5 **Mean and variance of observed resistance factors for ZDV and IDV mutants.**
(PDF)

Table S6 **Median of mechanistic waiting times predicted from 500 hybrid deterministic-stochastic simulations of the model for IDV monotherapy.**
(PDF)

Text S1 **Model descriptions, parameter sensitivity, pharmacokinetics and hybrid deterministic-stochastic simulations.**
(PDF)
