# Supplementary material for: Estimating HIV-1 Fitness Characteristics from Cross-Sectional Genotype Data
Source: PLoS Comput Biol. 2014 Nov 6;10(11):e1003886. doi: 10.1371/journal.pcbi.1003886 (PMC4222584; doi:10.1371/journal.pcbi.1003886)
Supplement: Figure S1 — Cumulative histograms of correlations of fitnesses between valid fits and best fit. (PDF) [file pcbi.1003886.s001.pdf]

Supporting Information: Estimating HIV-1 Fitness Characteristics from Cross-sectional Genotype Data

Sathej Gopalakrishnan, Hesam Montazeri, Stephan Menz, Niko Beerenwinkel, Wilhelm Huisinga

Supplementary Figure S1

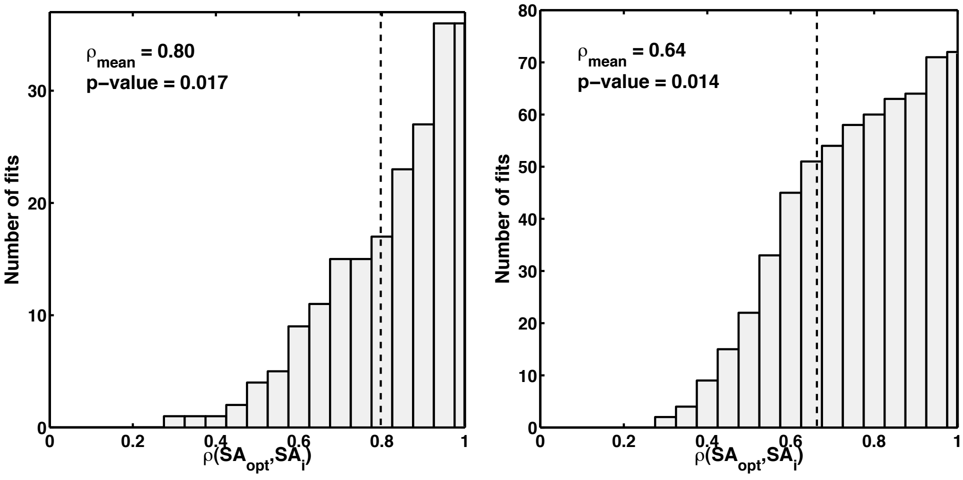

Cumulative histograms of correlations of fitnesses between valid fits and best fit. Cumulative histograms of Spearman rank correlation of estimated fitnesses of mutants (quantified by selective advantages) between the best fit and every other valid fit for ZDV mutants (left) and IDV mutants (right). The vertical dashed line shows the mean correlation.
