# Supplementary material for: Estimating HIV-1 Fitness Characteristics from Cross-Sectional Genotype Data
Source: PLoS Comput Biol. 2014 Nov 6;10(11):e1003886. doi: 10.1371/journal.pcbi.1003886 (PMC4222584; doi:10.1371/journal.pcbi.1003886)
Supplement: Figure S2 — Distribution of mechanistic waiting times from 1000 simulations of the virus dynamics model. (PDF) [file pcbi.1003886.s002.pdf]

Supporting Information: Estimating HIV-1 Fitness Characteristics from Cross-sectional Genotype Data

Sathej Gopalakrishnan, Hesam Montazeri, Stephan Menz, Niko Beerenwinkel, Wilhelm Huisinga

Supplementary Figure S2

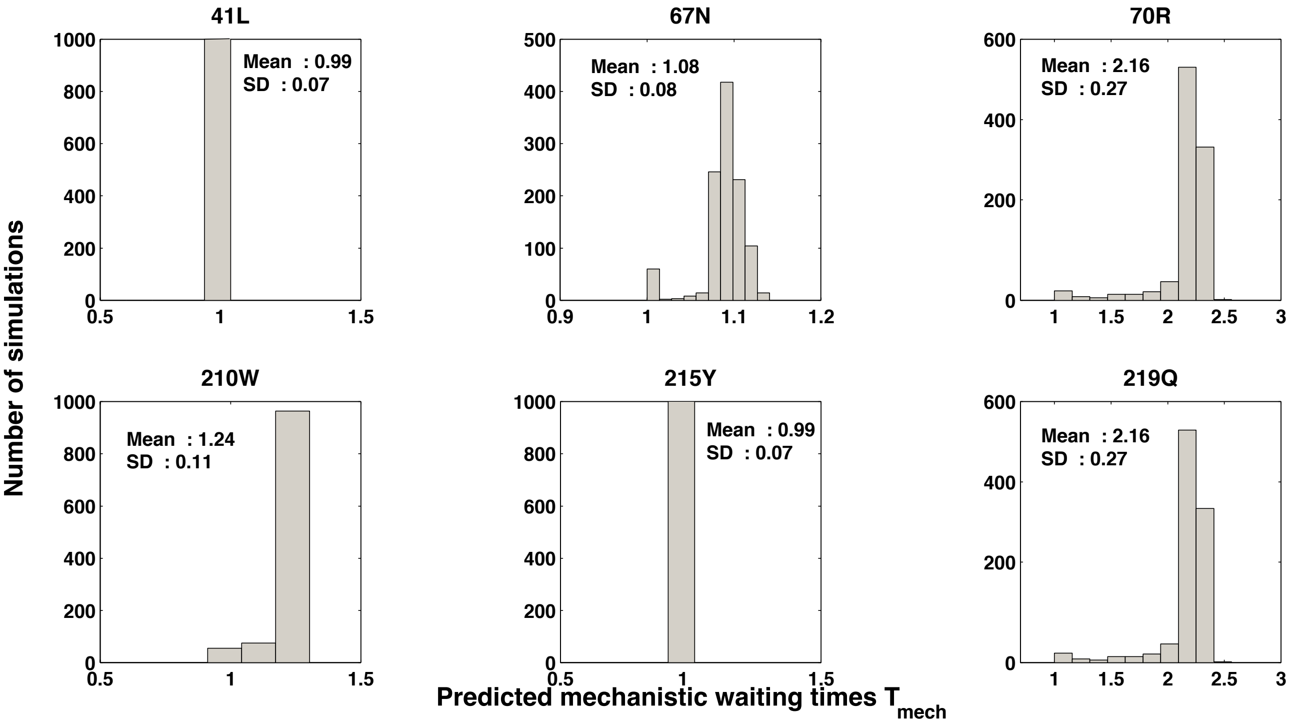

Distribution of mechanistic waiting times from 1000 simulations of the virus dynamics model. The turn-over parameters of the virus dynamics model (infection rate constants, death rate constants, clearances and other reaction rate constants) for ZDV therapy were perturbed by up to ±50% and 1000 simulations were performed by choosing parameters from a uniform distribution on this range (see section D of Supplementary Text S1 for details). The distribution of the waiting times to different mutations with their mean and standard deviation (SD) are shown. The predicted waiting times showed an excellent correlation (r = 0.98, p-value = 0.0006) with the average statistical waiting times used to fit the model.
