# Supplementary material for: Estimating HIV-1 Fitness Characteristics from Cross-Sectional Genotype Data
Source: PLoS Comput Biol. 2014 Nov 6;10(11):e1003886. doi: 10.1371/journal.pcbi.1003886 (PMC4222584; doi:10.1371/journal.pcbi.1003886)
Supplement: Figure S3 — Two compartment pharmacokinetic model for ZDV and fluctuating drug-effect. (PDF) [file pcbi.1003886.s003.pdf]

Supporting Information:
Estimating HIV-1 Fitness Characteristics from Cross-sectional Genotype Data

Sathej Gopalakrishnan, Hesam Montazeri, Stephan Menz, Niko Beerenwinkel, Wilhelm Huisinga

Supplementary Figure S3

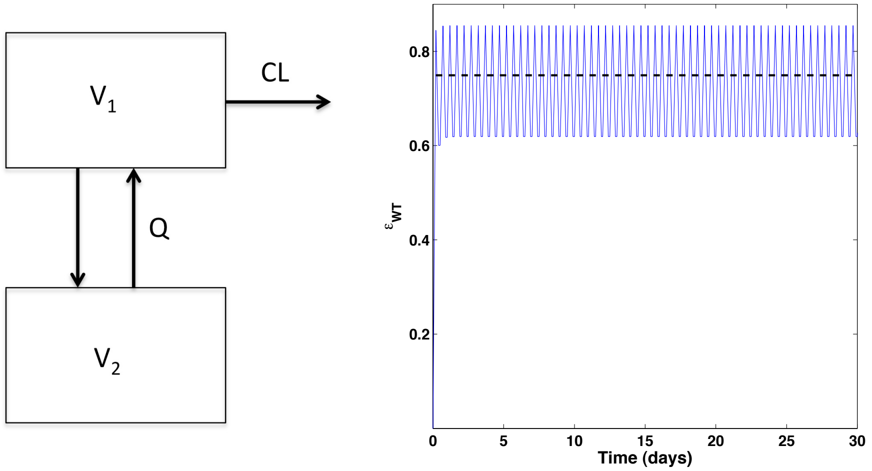

Two compartment pharmacokinetic model for ZDV and fluctuating drug-effect. **Left:** Two-compartment PK model for ZDV. Please see section E of Supplementary Text S1 for a description and the parameters used. **Right:** Fluctuations in ϵ_{WT} (contrast with constant drug effect, where we used $\epsilon_{\mathrm{WT}} = 0.75$ (dashed line)).
