# Supplementary material for: Estimating HIV-1 Fitness Characteristics from Cross-Sectional Genotype Data
Source: PLoS Comput Biol. 2014 Nov 6;10(11):e1003886. doi: 10.1371/journal.pcbi.1003886 (PMC4222584; doi:10.1371/journal.pcbi.1003886)
Supplement: Figure S4 — Variability of resistance factors under ZDV and IDV therapy. (PDF) [file pcbi.1003886.s004.pdf]

Supporting Information:
Estimating HIV-1 Fitness Characteristics from Cross-sectional Genotype Data

Sathej Gopalakrishnan, Hesam Montazeri, Stephan Menz, Niko Beerenwinkel, Wilhelm Huisinga

Supplementary Figure S4

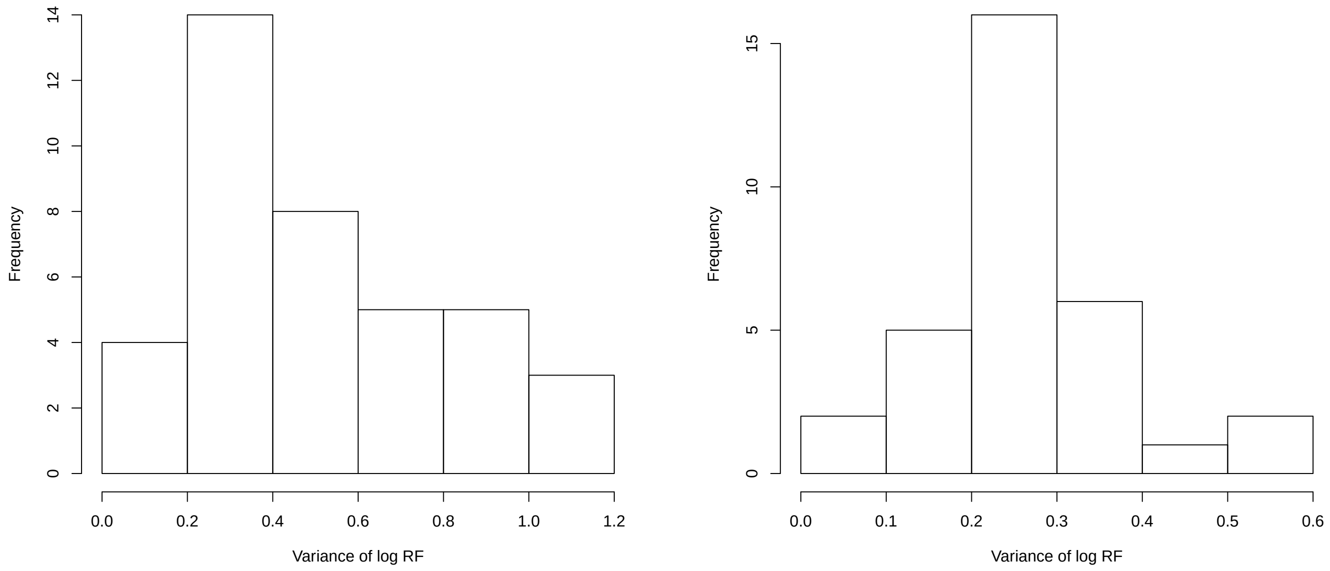

Variability of resistance factors under ZDV and IDV therapy. The histograms show the variability of measured resistance factors for different genotypes under ZDV (left) and IDV (right) therapy. Variances of resistance factors for the mutant genotypes in the ZDV and IDV genotype lattices are reported in Supplementary Table S5.
