# Supplementary material for: Estimating HIV-1 Fitness Characteristics from Cross-Sectional Genotype Data
Source: PLoS Comput Biol. 2014 Nov 6;10(11):e1003886. doi: 10.1371/journal.pcbi.1003886 (PMC4222584; doi:10.1371/journal.pcbi.1003886)
Supplement: Figure S5 — Predicted mutational abundance from 500 hybrid deterministic-stochastic simulations of IDV monotherapy. (PDF) [file pcbi.1003886.s005.pdf]

Supporting Information: Estimating HIV-1 Fitness Characteristics from Cross-sectional Genotype Data

Sathej Gopalakrishnan, Hesam Montazeri, Stephan Menz, Niko Beerenwinkel, Wilhelm Huisinga

Supplementary Figure S5

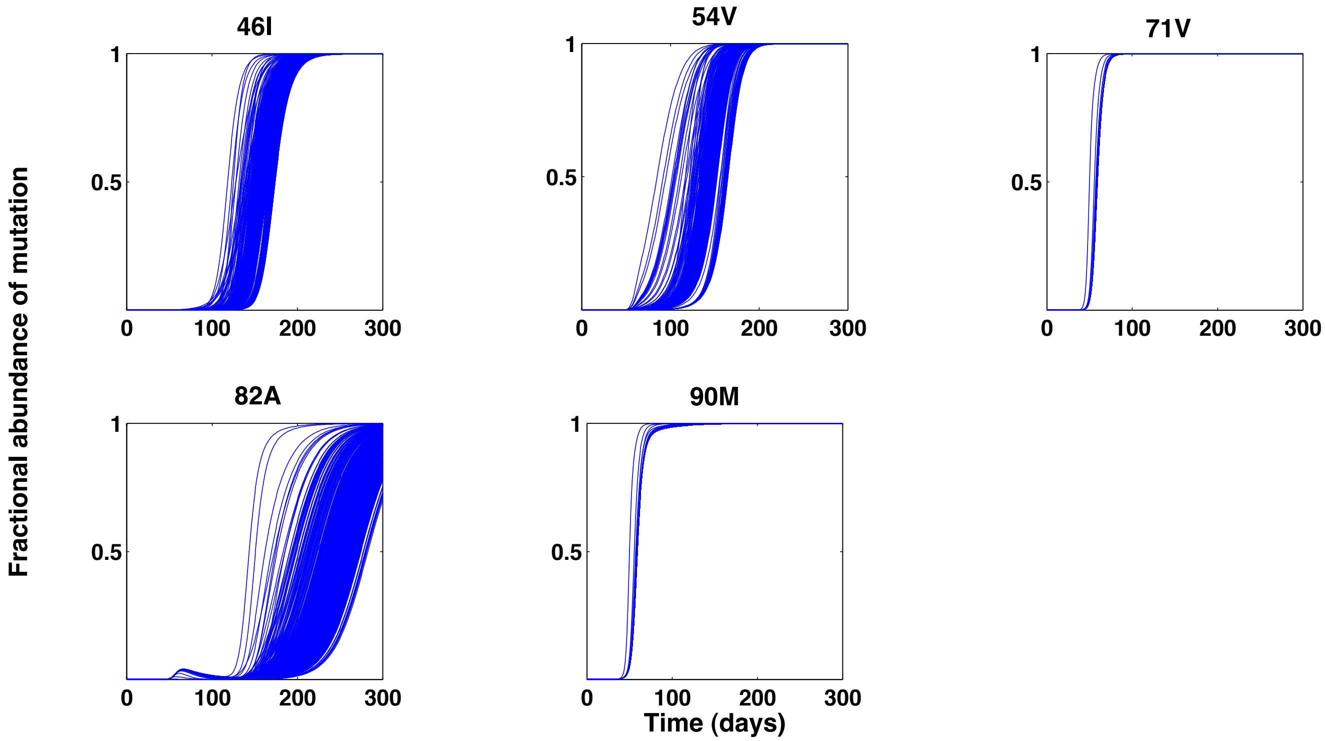

Predicted mutational abundance from 500 hybrid deterministic-stochastic simulations of IDV monotherapy. The model for IDV monotherapy was simulated using a hybrid deterministic-stochastic algorithm to compute 500 realizations (see Supplementary Text S1). The predicted fractional abundance of different mutations are shown. The median of the mechanistic waiting times to the mutations correlated well with the statistical average waiting times (r = 0.98, p-value = 0.0006, see Supplementary Table S6).
