# Supplementary material for: Estimating HIV-1 Fitness Characteristics from Cross-Sectional Genotype Data
Source: PLoS Comput Biol. 2014 Nov 6;10(11):e1003886. doi: 10.1371/journal.pcbi.1003886 (PMC4222584; doi:10.1371/journal.pcbi.1003886)
Supplement: Figure S6 — Cumulative histograms of correlations of fitness costs between fits with varying RFs and the best fit. (PDF) [file pcbi.1003886.s006.pdf]

Supporting Information:
Estimating HIV-1 Fitness Characteristics from Cross-sectional Genotype Data

Sathej Gopalakrishnan, Hesam Montazeri, Stephan Menz, Niko Beerenwinkel, Wilhelm Huisinga

Supplementary Figure S6

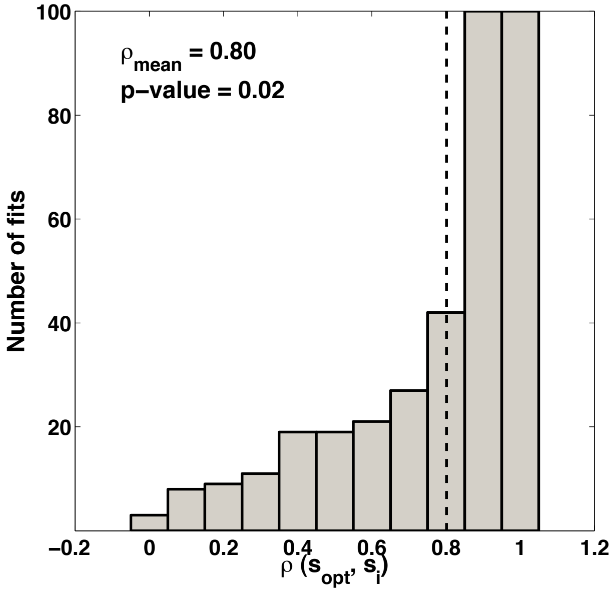

Cumulative histograms of correlations of fitness costs between fits with varying RFs and the best fit. A cumulative histogram that shows the rank correlation between fitness costs in the best fit and those estimated by varying RFs in a certain range (refer section D of Supplementary Text S1 for description). The estimated fitness costs show a strong and statistically significant average correlation ($\rho = 0.80$, p-value $= 0.02$), in spite of the fact that only one round of estimation was performed for each set of RF (in view of computational costs).
