# Supplementary material for: Estimating HIV-1 Fitness Characteristics from Cross-Sectional Genotype Data
Source: PLoS Comput Biol. 2014 Nov 6;10(11):e1003886. doi: 10.1371/journal.pcbi.1003886 (PMC4222584; doi:10.1371/journal.pcbi.1003886)
Supplement: Table S1 — Parameters of viral dynamics model used in the simulations. (PDF) [file pcbi.1003886.s007.pdf]

# Supporting Information: Estimating HIV-1 Fitness Characteristics from Cross-sectional Genotype Data

Sathej Gopalakrishnan, Hesam Montazeri, Stephan Menz, Niko Beerenwinkel, Wilhelm Huisinga

## Supplementary Table S1

**Parameters of viral dynamics model used in the simulations**

| Parameter | Description | Value |
|---|---|---|
| $\lambda_{\mathrm{TU}}$ | Synthesis of uninfected target T-cells | $2{\cdot}10^{9}$ |
| $\beta_{\mathrm{T}}$ | Infection of T-cells | $8{\cdot}10^{-12}$ |
| $\widetilde{\mathrm{N_T}} = \mathrm{N_{TI}} + \mathrm{N_{TNI}}$ | Viral production from infected T-cells | 1000 |
| $\delta_{\mathrm{TU}}, \delta_{\mathrm{T1}}$ | Death of uninfected and early stage infected T-cells | 0.02 |
| $\delta_{\mathrm{T2}}$ | Death of late stage infected T-cells | 1 |
| $\mathrm{CL_V}$ | Viral clearance | 23 |
| $\rho_{\mathrm{PR},\phi}$ | Probability of successful assembly and maturation of viruses | 0.67 |
| $\rho_{\mathrm{rev},\phi}$ | Probability of successful reverse transcription | 0.33 |
| $\mu$ | Basal mutation | $3.4 \cdot 10^{-5}$ |
| $\mathrm{k_T}$ | Integration of viral DNA in T-cells | 0.35 |
| $\delta_{\mathrm{PIC},T}$ | Clearance of pre-integration complex in T-cells | 0.35 |
| $\alpha$ | Activation of latent T-cells | $10^{-3}$ |
| $\lambda_{\mathrm{MU}}$ | Synthesis of uninfected target macrophages | $6.9 \cdot 10^{7}$ |
| $\delta_{\mathrm{MU}}, \delta_{\mathrm{M1}}$ | Death of uninfected and early stage infected macrophages | 0.0069 |
| $\delta_{\mathrm{M2}}$ | Death of late stage infected macrophages | 0.09 |
| $\delta_{\mathrm{TL}}$ | Death of latent T-cells | $10^{-4}$ |
| $\delta_{\mathrm{PIC,M}}$ | Clearance of pre-integration complex in macrophages | 0.0035 |
| $p$ | Probability of entering latency | $8 \cdot 10^{-6}$ |
| $\mathrm{k_M}$ | Integration of viral DNA in macrophages | 0.07 |
| $\beta_M$ | Infection of macrophages | $10^{-14}$ |
| $\widetilde{\mathrm{N_M}} = \mathrm{N_{MI}} + \mathrm{N_{MNI}}$ | Viral production from infected macrophages | 100 |

All parameters in units [1/day], except $\rho_{\mathrm{PR},\phi}$ and $\rho_{\mathrm{rev},\phi}$ (unitless) and $\mu$ in [1/(rev. trans · base)]. The references to all parameter values are provided in [1].

## References

1. von Kleist M, Menz S, Stocker H, Arasteh K, Schütte C, et al. (2011) HIV quasispecies dynamics during pro-active treatment switching: impact on multi-drug resistance and resistance archiving in latent reservoirs. PLoS One 8: e18204.
