# Supplementary material for: Estimating HIV-1 Fitness Characteristics from Cross-Sectional Genotype Data
Source: PLoS Comput Biol. 2014 Nov 6;10(11):e1003886. doi: 10.1371/journal.pcbi.1003886 (PMC4222584; doi:10.1371/journal.pcbi.1003886)
Supplement: Table S2 — Statistical and mechanistic waiting times to observe mutations under ZDV and IDV monotherapy. (PDF) [file pcbi.1003886.s008.pdf]

Supporting Information: Estimating HIV-1 Fitness Characteristics from Cross-sectional Genotype Data

Sathej Gopalakrishnan, Hesam Montazeri, Stephan Menz, Niko Beerenwinkel, Wilhelm Huisinga

Supplementary Table S2

Statistical and mechanistic waiting times to observe mutations under ZDV and IDV monotherapy.

Drug	Mutation (e)	Normalized statistical mutation rates (λ_e/λ_s)	Statistical average waiting times	Mechanistic waiting times
ZDV	41L	0.81 (0.73, 0.88)	1.00	1.00
	67N	0.73 (0.68, 0.82)	1.10	1.10
	70R	0.75 (0.63, 0.87)	2.18	2.26
	210W	4.11 (3.29, 4.96)	1.28	1.29
	215Y	9.19 (6.50, 17.17)	1.08	1.01
	219Q	5.14 (3.77, 8.80)	2.34	2.26
IDV	46I	1.49 (1.20, 1.84)	1.47	1.53
	54V	2.03 (1.68, 2.61)	1.36	1.44
	71V	0.69 (0.62, 0.76)	1.02	1.00
	82A	1.13 (0.92, 1.44)	1.64	1.71
	90M	0.70 (0.62, 0.76)	1.00	1.00

The statistically estimated rates of occurrence of different mutations (column 3) with 95% confidence intervals in parentheses, the average statistical waiting times (column 4) and the corresponding mechanistic waiting times (column 5) to observe mutations at the different positions under ZDV and IDV monotherapy. The average statistical and mechanistic waiting times were calculated as described in the *Methods* section. Both waiting times are expressed relative to the time to the fastest occurring mutation.
