# Supplementary material for: Estimating HIV-1 Fitness Characteristics from Cross-Sectional Genotype Data
Source: PLoS Comput Biol. 2014 Nov 6;10(11):e1003886. doi: 10.1371/journal.pcbi.1003886 (PMC4222584; doi:10.1371/journal.pcbi.1003886)
Supplement: Table S3 — Conservation of estimated fitness characteristics of ZDV and IDV mutants amongst valid fits. (PDF) [file pcbi.1003886.s009.pdf]

Supporting Information: Estimating HIV-1 Fitness Characteristics from Cross-sectional Genotype Data

Sathej Gopalakrishnan, Hesam Montazeri, Stephan Menz, Niko Beerenwinkel, Wilhelm Huisinga

Supplementary Table S3

Conservation of estimated fitness characteristics of ZDV and IDV mutants amongst valid fits.

Drug	Observation	% of valid fits where conserved
ZDV	Average fitness cost of TAM-1 mutants < Average fitness cost of TAM-2 mutants	90
	Average SA of TAM-1 mutants > Average SA of TAM-2 mutants	100
	s({41L, 210W, 215Y}) > s({41L, 215Y})	92
	s({67N,70R}) > s({67N})	80
	s({41L, 210W, 215Y}) < s({67N, 70R, 219Q})	81
	SA({41L, 210W, 215Y}) > SA({41L, 215Y})	64
IDV	s({90M}) < s({71V})	65
	71V compensates fitness	77
	SA({71V, 82A}) ≈ SA({54V, 71V, 82A})	71

Here, s denotes the fitness cost and SA the selective advantage.
