# Supplementary material for: Estimating HIV-1 Fitness Characteristics from Cross-Sectional Genotype Data
Source: PLoS Comput Biol. 2014 Nov 6;10(11):e1003886. doi: 10.1371/journal.pcbi.1003886 (PMC4222584; doi:10.1371/journal.pcbi.1003886)
Supplement: Table S4 — Statistical and mechanistic waiting times to observe mutations under ZDV monotherapy with ZDV concentration described by a two-compartment pharmacokinetic model. (PDF) [file pcbi.1003886.s010.pdf]

Supporting Information:
Estimating HIV-1 Fitness Characteristics from Cross-sectional Genotype Data

Sathej Gopalakrishnan, Hesam Montazeri, Stephan Menz, Niko Beerenwinkel, Wilhelm Huisinga

Supplementary Table S4

Statistical and mechanistic waiting times to observe mutations under ZDV monotherapy with ZDV concentration described by a two-compartment pharmacokinetic model.

Mutation (e)	Statistical average waiting times	Mechanistic waiting times
41L	1.00	1.00
67N	1.10	1.06
70R	2.18	1.81
210W	1.28	1.10
215Y	1.08	1.02
219Q	2.34	1.81

The average statistical waiting times and the corresponding mechanistic waiting times to observe mutations at the different positions under ZDV monotherapy. ZDV concentration was assumed to be described by a two-compartment PK model (see section E of Supplementary Text S1 and Supplementary Figure S3). The average statistical and mechanistic waiting times are calculated as described in the *Methods* section. Both waiting times are expressed relative to the time to the fastest occurring mutation. The predicted mechanistic waiting times had a high and significant correlation (r = 0.99, p-value = 0.0001) with the statistical waiting times.
