# Supplementary material for: Estimating HIV-1 Fitness Characteristics from Cross-Sectional Genotype Data
Source: PLoS Comput Biol. 2014 Nov 6;10(11):e1003886. doi: 10.1371/journal.pcbi.1003886 (PMC4222584; doi:10.1371/journal.pcbi.1003886)
Supplement: Table S5 — Mean and variance of observed resistance factors for ZDV and IDV mutants. (PDF) [file pcbi.1003886.s011.pdf]

Supporting Information:
Estimating HIV-1 Fitness Characteristics from Cross-sectional Genotype Data

Sathej Gopalakrishnan, Hesam Montazeri, Stephan Menz, Niko Beerenwinkel, Wilhelm Huisinga

Supplementary Table S5

Mean and variance of observed resistance factors for ZDV and IDV mutants.

Drug	Mutant	#observations	obs. avg. log RF	obs. var. log RF
ZDV	{67N}	9	0.313	0.632
	{67N, 70R}	44	0.754	0.519
	{67N, 70R, 219Q}	104	1.059	0.530
	{41L}	27	0.297	0.303
	{41L, 215Y}	83	0.618	0.308
	{41L, 210W, 215Y}	137	1.068	0.479
	{41L, 67N, 210W, 215Y}	231	1.363	0.569
	{41L, 67N, 70R, 210W, 215Y, 219Q}	9	2.074	1.190
IDV	{90M}	114	0.553	0.323
	{71V}	60	0.188	0.281
	{46I, 90M}	113	1.147	0.215
	{71V, 90M}	113	0.980	0.220
	{54V, 71V}	40	1.352	0.240
	{71V, 82A}	41	1.496	0.361
	{54V, 71V, 82A}	76	1.279	0.217
	{54V, 71V, 90M}	73	1.468	0.254
	{71V, 82A, 90M}	26	1.392	0.180
	{46I, 71V, 90M}	100	1.269	0.202
	{54V, 71V, 82A, 90M}	101	1.377	0.163
	{46I, 71V, 82A, 90M}	33	1.498	0.194
	{46I, 54V, 71V, 90M}	62	1.622	0.206
	{46I, 54V, 71V, 82A, 90M}	51	1.490	0.093

Sample mean and variance of observed resistance factors (on a logarithmic scale, log RF) reported for mutants arising during ZDV and IDV therapy.
