# Supplementary material for: Estimating HIV-1 Fitness Characteristics from Cross-Sectional Genotype Data
Source: PLoS Comput Biol. 2014 Nov 6;10(11):e1003886. doi: 10.1371/journal.pcbi.1003886 (PMC4222584; doi:10.1371/journal.pcbi.1003886)
Supplement: Table S6 — Median of mechanistic waiting times predicted from 500 hybrid deterministic-stochastic simulations of the model for IDV monotherapy. (PDF) [file pcbi.1003886.s012.pdf]

Supporting Information: Estimating HIV-1 Fitness Characteristics from Cross-sectional Genotype Data

Sathej Gopalakrishnan, Hesam Montazeri, Stephan Menz, Niko Beerenwinkel, Wilhelm Huisinga

Supplementary Table S6

Median of mechanistic waiting times predicted from 500 hybrid deterministic-stochastic simulations of the model for IDV monotherapy.

Mutation (e)	Statistical average waiting times	Median of mechanistic waiting times
46I	1.47	2.69
54V	1.36	2.42
71V	1.02	1.01
82A	1.64	4.01
90M	1.00	1.00

The statistical waiting times and median mechanistic waiting times derived from 500 hybrid deterministic-stochastic realizations of the model for IDV monotherapy are compared (see section F of Supplementary Text S1, Supplementary Figure S5). The predicted mechanistic waiting times continued to correlate well with the statistical waiting times (r = 0.98, p-value = 0.0006).
