# Supplementary material for: Estimating HIV-1 Fitness Characteristics from Cross-Sectional Genotype Data
Source: PLoS Comput Biol. 2014 Nov 6;10(11):e1003886. doi: 10.1371/journal.pcbi.1003886 (PMC4222584; doi:10.1371/journal.pcbi.1003886)
Supplement: Text S1 — Model descriptions, parameter sensitivity, pharmacokinetics and hybrid deterministic-stochastic simulations. (PDF) [file pcbi.1003886.s013.pdf]

Supporting Information: Estimating HIV-1 Fitness Characteristics from Cross-sectional Genotype Data

Sathej Gopalakrishnan, Hesam Montazeri, Stephan Menz, Niko Beerenwinkel, Wilhelm Huisinga

Supplementary Text S1

Model descriptions, parameter sensitivity, pharmacokinetics and hybrid deterministic-stochastic simulations

A. The ODE formulation of the mechanistic model

The system of ODEs governing the viral dynamics model is provided below. See [1] for a detailed description of the model. Here, we provide the ODEs to illustrate the integration of the mutation scheme into the mechanistic model:

$$
\begin{aligned}
\frac{\mathrm{d}}{\mathrm{d}t}\mathrm{TU} &= \lambda_{\mathrm{TU}} - \delta_{\mathrm{TU}} \cdot \mathrm{TU} + \sum_{g \in \mathcal{G}} \delta_{\mathrm{PIC,T}} \cdot \mathrm{T}_{1,g} - \sum_{g \in \mathcal{G}} \beta_{\mathrm{T},g} \cdot \mathrm{V}_{\mathrm{I},g} \cdot \mathrm{TU} \\
\frac{\mathrm{d}}{\mathrm{d}t}\mathrm{MU} &= \lambda_{\mathrm{MU}} - \delta_{\mathrm{MU}} \cdot \mathrm{MU} + \sum_{g \in \mathcal{G}} \delta_{\mathrm{PIC,M}} \cdot \mathrm{M}_{1,g} - \sum_{g \in \mathcal{G}} \beta_{\mathrm{M},g} \cdot \mathrm{V}_{\mathrm{I},g} \cdot \mathrm{MU} \\
\frac{\mathrm{d}}{\mathrm{d}t}\mathrm{T}_{1,g} &= \beta_{\mathrm{T},g} \cdot \mathrm{V}_{\mathrm{I},g} \cdot \mathrm{TU} - \big(\delta_{\mathrm{T}_1} + \delta_{\mathrm{PIC,T}} + k_{\mathrm{T},g}\big) \cdot \mathrm{T}_{1,g} \\
\frac{\mathrm{d}}{\mathrm{d}t}\mathrm{M}_{1,g} &= \beta_{\mathrm{M,g}} \cdot \mathrm{V}_{\mathrm{I},g} \cdot \mathrm{MU} - \big(\delta_{\mathrm{M}_1} + \delta_{\mathrm{PIC,M}} + k_{\mathrm{M},g}\big) \cdot \mathrm{M}_{1,g} \\
\frac{\mathrm{d}}{\mathrm{d}t}\mathrm{T}_{2,g} &= \sum_{g' \in \mathcal{G}} (1-p) \cdot k_{\mathrm{T},g'} \cdot \mathrm{T}_{1,g'} \cdot r_{g' \to g} + \alpha \cdot \mathrm{T}_{\mathrm{L},g} - \delta_{\mathrm{T}_2} \cdot \mathrm{T}_{2,g} \\
\frac{\mathrm{d}}{\mathrm{d}t}\mathrm{M}_{2,g} &= \sum_{g' \in \mathcal{G}} k_{\mathrm{M},g'} \cdot \mathrm{M}_{1,g'} \cdot r_{g' \to g} - \delta_{\mathrm{M}_2} \cdot \mathrm{M}_{2,g} \\
\frac{\mathrm{d}}{\mathrm{d}t}\mathrm{T}_{\mathrm{L},g} &= \sum_{g' \in \mathcal{G}} p \cdot k_{\mathrm{T},g'} \cdot \mathrm{T}_{1,g'} \cdot r_{g' \to g} - \alpha \cdot \mathrm{T}_{\mathrm{L},g} - \delta_{\mathrm{TL}} \cdot \mathrm{T}_{\mathrm{L},g} \\
\frac{\mathrm{d}}{\mathrm{d}t}\mathrm{V}_{\mathrm{I,g}} &= \mathrm{N}_{\mathrm{TI},g} \cdot \mathrm{T}_{2,g} + \mathrm{N}_{\mathrm{MI},g} \cdot \mathrm{M}_{2,g} - \Big[\mathrm{CL}_{\mathrm{V}} + \big(\mathrm{CL}_{\mathrm{T},g} + \beta_{\mathrm{T},g}\big)\mathrm{TU} + \big(\mathrm{CL}_{\mathrm{M},g} + \beta_{\mathrm{M},g}\big)\mathrm{MU}\Big] \cdot \mathrm{V}_{\mathrm{I},g} \\
\frac{\mathrm{d}}{\mathrm{d}t}\mathrm{V}_{\mathrm{NI,g}} &= \mathrm{N}_{\mathrm{TNI},g} \cdot \mathrm{T}_{2,g} + \mathrm{N}_{\mathrm{MNI},g} \cdot \mathrm{M}_{2,g} - \mathrm{CL}_{\mathrm{V}} \cdot \mathrm{V}_{\mathrm{NI,g}}
\end{aligned}
$$

where the subscript g refers to a mutant genotype g from the genotype lattice $\mathcal{G}$. We modelled mutations as occurring between stages T_1 and T_2 of the infection cycle. We embedded the genotype lattice $\mathcal{G}$ into the system of ODEs and enabled mutation reactions compatible with $\mathcal{G}$. We considered only those mutation events where the number of amino acid sites at which the mutant genotypes involved differ was one or two. This is owing to the fact that, though multiple nucleotide changes are simultaneously possible, the probability of such events is very low. The probability of mutation from genotype g' to g

was written as

$$r_{g' \to g} = \begin{cases} \mu & \text{; single mutation} \\ \frac{1}{2}\mu & \text{; double mutation} \end{cases} \tag{1}$$

Under the assumption that mutations occur independently of each other (which is plausible for mutation sites that are apart from each other in the viral genome) one would expect a μ^2 term for a double mutation (at the nucleotide level) in a single round of transcription. A double mutation could alternatively occur via one of the two intermediate single-mutation genotypes. These, however, we do not explicitly take into account in our model. Depending on the viral level of these intermediate genotypes, the probability of a double-mutation occurring via a single-mutation intermediate can be expected to be much larger (like it is much simpler to roll one 'six' and continue to roll until the second 'six'—expected number of throws $2/p = 12$ with $p = 1/6$— than to roll two 'six' in a row —expected number of throws $1/p^2 = 36$). In addition, the probability of mutation might differ between distant mutations and neighbouring mutations. In the absence of knowledge on the viral levels of intermediate genotypes and on conditional mutation rates, we used the probabilities of mutations in eq. (1). Reverse transcriptase inhibitors such as ZDV inhibit the infection rates β_T, β_M and the rates of clearance due to unsuccessful infection CL_T and CL_M, while protease inhibitors such as IDV inhibit the viral production rates N_{TI} and N_{MI}. We parametrized the model as in [1]. Supplementary Table S1 gives the parameter values used for simulations.

B. Statistical and mechanistic waiting times

Our estimated fitness costs resulted in mechanistic waiting times that matched very well with the average statistical waiting times for both ZDV (Pearson coefficient of correlation r = 0.99, p = 0.0001) and IDV (Pearson coefficient of correlation r = 0.99, p = 0.001), see Supplementary Table S2. This served as a good first plausibility check, as the mechanistic model can be regarded as a more detailed version of the statistical mutation scheme, as far as the prediction of waiting times to different mutations is concerned.

C. Parameter estimation and identifiability

To address concerns of parameter identifiability, we performed $N = 500$ rounds of estimation starting from different randomly chosen initial estimates. For both ZDV and IDV, we considered all fits with an RMSD < 0.1 between the statistical and mechanistic waiting times as equally valid. The average error in the normalized waiting times estimated from clinical data was ±10% and hence we chose this threshold for the RMSD. This is because all fits with an RMSD less than the error incurred in the data used for estimating fitness costs cannot essentially be distinguished. This is in accordance with similar approaches adopted earlier for parameter estimation in detailed mechanistic models [2]. The RMSD for a fit f was defined as follows:

$$\mathrm{RMSD}_f = \sqrt{\frac{1}{n} \cdot \sum_{e \in \mathcal{E}} (\mathbb{E}[T_{e,\mathrm{stat}}] - T_{e,\mathrm{mech}})^2}, \tag{2}$$

where e denotes a mutation occurring in the corresponding poset $\mathcal{E}$ and n denotes the total number of mutations considered.

Of the $N = 500$ fits, we recovered 35 valid fits for ZDV and 72 valid fits for IDV. For ZDV mutants, we noted a strong and significant Spearman rank correlation ($\rho_{\mathrm{mean}} = 0.80$, p-value = 0.017) of selective advantages between the best fit and all other valid fits. Similarly for IDV mutants, the correlation was 0.64 (p-value = 0.014). This indicated that the order of fitness characteristics that we estimated was strongly preserved across all the considered fits and enabled us to make valid inferences from our fitness estimates. We also examined specific observations on fitness characteristics for their behavior across valid fits and observed that they were strongly conserved (see Supplementary Figure S1 and Supplementary Table S3).

D. Sensitivity analysis of model predictions with respect to parameters of viral dynamics model

Further, since the turn-over parameters of the virus dynamics model are subject to uncertainty, we performed a sensitivity analysis of our predictions of mechanistic waiting times to different mutations. We used our example of ZDV therapy to illustrate this. To this end, we chose each turn-over parameter (the death rates of the different stages, the infection rates, the rates of integration of viral DNA and clearance of the infectious and non-infectious viruses) from a uniform distribution with a range of up-to $\pm 50\%$ of their original value. Note that this is certainly a large enough range for these parameters (compared to 20-25% range reported for viral clearance and death rates of infected cells in [3], 5-10% standard errors reported in [4] or 2-12% standard errors estimated in [5]). The distribution of predicted waiting times (see Supplementary Figure S2) had an excellent correlation (Pearson coefficient of correlation $r = 0.98$, p-value $= 0.0006$) with the average statistical times used to fit the model. This showed our predictions to be robust with respect to variations in the parameters of the virus dynamics model.

Additionally, since our estimation of fitness characteristics is a two-stage process (involving estimation of resistance factors RF, followed by estimation of fitness costs), we also tested the impact of variability of the first step on the second. To accomplish this, we chose 100 different sets of RFs for ZDV resistant mutants by sampling from the estimated distributions (estimation of RFs by isotonic regression assumes a normal distribution, as described in reference [16] of the main text). We then re-estimated fitness costs using each of these sets of RFs. We observed that the ranking of estimated fitness costs of the different mutants remained conserved (Supplementary Figure S6). For each of these sets of RFs, we performed only one round of estimation (in view of computational costs). In spite of this, the correlation between the estimated fitness costs in each set and the best fit was strong and statistically significant ($\rho = 0.80$, p-value $= 0.02$). This showed that the impact of variability in the first stage of estimation does not extend to the conclusions we make in the article.

E. Integration of pharmacokinetic profiles

While we assumed a constant drug effect (ϵ_{WT}), as is commonly done in viral dynamics models [6–8], the integration of pharmacokinetic (PK) drug profiles can also be realized in our model. To illustrate how this can be performed, we considered a two-compartment PK model of ZDV (Supplementary Figure S3) with a central volume of distribution V_1 and peripheral volume of distribution V_2. The drug was linearly cleared from the central compartment with a clearance CL, and the inter-compartmental flow was Q. The model is specified by the coupled linear system of ODEs given by eq. (3). More detailed physiologically based PK models have been proposed [9], but this is beyond the scope of the current work. The goal here was to both demonstrate the integration of PK models into our framework and to assess its impact on our model predictions.

$$
\begin{aligned}
V_1 \frac{d}{dt} C_1 &= Q(C_2 - C_1) - \mathrm{CL} \cdot C_1 \\
V_2 \frac{d}{dt} C_2 &= Q(C_1 - C_2)
\end{aligned} \tag{3}
$$

with initial conditions $C_1(0) = \mathrm{Dose}/V_1$ and $C_2(0) = 0$.

We set the PK parameters $\mathrm{CL} = 1.6\ L/h/kg$ and $V_1 = 1.6\ L/kg$ for ZDV from literature (see [9], [10]). We chose parameters $Q = 2 \cdot CL$ and $V_2 = 2 \cdot V_1$ so as to obtain a steady-state peak-to-trough ratio in the peripheral (effect) compartment of about 2-3, in line with previous observations for NRTIs [9, 11]. Our simulations were scaled for an individual with a weight of 70 kg with a dose of 300 mg every 12 hours. The model was described by ODEs (see [12] for more details on such models). Note that, in principle, to model the influence of drug concentrations on antiviral effects, we need access to intracellular concentrations of the active metabolite of the drug, a quantity that is seldom available. Nevertheless, a simple PK

model such as the current one serves to illustrate the point. The concentration in the peripheral (effect) compartment was coupled to the virus dynamics model via eq.(1) in the article. The parameters β_{T}, $\mathrm{CL_T}$, β_{M} and $\mathrm{CL_M}$, influenced by ZDV were now functions of time. We observed that the predicted waiting times did not vary significantly (Supplementary Table S4) from the constant drug-concentration case. The predicted mechanistic waiting times retained a high correlation with the average statistical waiting times ($r = 0.99$, p-value $= 0.0001$).The order of appearance of mutations was also preserved. This is mainly owing to the fact that ZDV monotherapy is generally ineffective and fluctuating drug concentrations (within a meaningful range) would not deter the occurrence of mutations. With combination therapy and drug-interactions, the behaviour is expected to be more complex. Recent work by Rosenbloom et al [13] proposes the existence of mutant selection windows and studies the impact of drug concentrations on mutant selection. However, this work relied on mono-exponential PK profiles combined with *in vitro* IC50 measurements. More detailed investigations into the influence of PK on mutant selection are certainly warranted.

F. Hybrid deterministic-stochastic simulations of IDV monotherapy

Evolution of viral mutant genotypes is dictated by random events. The impact of stochastic modelling approaches can be expected to be larger for drugs with large genetic barriers (the genetic barrier to a drug is the number of mutations needed to confer high level resistance (>10-fold) to it). However, the drugs we considered have a low genetic barrier ($\sim$2). To assess the impact of stochastic approaches, we performed hybrid deterministic-stochastic simulations of our model, where rare mutation events were modelled stochastically, while e.g., the viral infection dynamics of abundant genotypes was modelled deterministically (a fully stochastic simulation would hardly be feasible even on supercomputers). The hybrid algorithm used is discussed in [14] that provides both the mathematical basis and the implementation scheme; in the hybrid model, a threshold criterion on the reaction propensity is used to determine whether a reaction is modelled stochastically or deterministically.

For illustration, we chose IDV since it is a more effective drug than ZDV in that it drives the viral load to lower levels. We chose a reaction propensity threshold of 10^{-3} for the deterministic-stochastic switch (which is reasonable, considering the much lower mutation rates of HIV) and computed 500 realizations of our model. The predicted mechanistic waiting times continued to agree well (details in Supplementary Table S6 and Supplementary Figure S5) with the average statistical waiting times ($r = 0.98$, p-value $= 0.0006$). We observed a delay in the appearance of certain mutations, in particular the later mutations. This is in line with similar observations made earlier [15].

A hybrid estimation procedure is possible, in principle, but suffers from hugely unrealistic computation times: The total run-time scales with the number of realization of the stochastic model; hence, even with only 500 realizations, the run-time would be a factor 500 larger than for the deterministic model, resulting in ca. a month!). Our model utilizes a deterministic framework and thus is an approximation. However, for the examples we considered, the model predictions of fitness costs, resistance factors, selective advantages and other features of viral dynamics agree very well with experiments. The design of efficient hybrid stochastic-deterministic approaches is currently under investigation.

References

1. von Kleist M, Menz S, Stocker H, Arasteh K, Schütte C, et al. (2011) HIV quasispecies dynamics during pro-active treatment switching: impact on multi-drug resistance and resistance archiving in latent reservoirs. PLoS One 8: e18204.

2. Chen WW, Schoeberl B, Jasper PJ, Niepel M, Nielsen UB, et al. (2009) Input-output behavior of ErbB signaling pathways as revealed by a mass action model trained against dynamic data. Mol Syst Biol 5: 239.

3. Perelson AS, Neumann AU, Markowitz M, Leonard JM, Ho DD (1996) HIV-1 dynamics in vivo: Virion clearance rate, infected cell life-span, and viral generation time. Science 271: 1582–1586.

4. Wu H, Ding AA, De Gruttola V (1998) Estimation of HIV dynamic parameters. Statistics in Medicine 17: 2463–2485.

5. Huang Y, Liu D, Wu H (2006) Hierarchical bayesian methods for estimation of parameters in a longitudinal HIV dynamic system. Biometrics 62: 413–423.

6. Perelson AS, Essunger P, Cao Y, Vesanen M, Hurley A, et al. (1997) Decay characteristics of HIV-1-infected compartments during combination therapy. Nature 387: 188–191.

7. Sedaghat AR, Dinoso JB, Shen L, Wilke CO, Siliciano RF (2008) Decay dynamics of HIV-1 depend on the inhibited stages of the viral life cycle. Proc Natl Acad Sci U S A 105: 4832–4837.

8. von Kleist M, Menz S, Huisinga W (2010) Drug-class specific impact of antivirals on the reproductive capacity of HIV. PLoS Comput Biol 6: e1000720.

9. von Kleist M, Huisinga W (2009) Pharmacokinetic–pharmacodynamic relationship of NRTIs and its connection to viral escape: An example based on zidovudine. European Journal of Pharmaceutical Sciences 36: 532–543.

10. Law V, Knox C, Djoumbou Y, Jewison T, Guo AC, et al. (2014) DrugBank 4.0: shedding new light on drug metabolism. Nucleic Acids Res 42.

11. Yuen GJ, Lou Y, Bumgarner NF, Bishop JP, Smith GA, et al. (2004) Equivalent steady-state pharmacokinetics of lamivudine in plasma and lamivudine triphosphate within cells following administration of lamivudine at 300 milligrams once daily and 150 milligrams twice daily. Antimicrob Agents Chemother 48: 176–182.

12. Bonate PL (2011) Pharmacokinetic-Pharmacodynamic Modeling and Simulation. Springer Science & Business Media.

13. Rosenbloom DI, Hill AL, Rabi SA, Siliciano RF, Nowak MA (2012) Antiretroviral dynamics determines HIV evolution and predicts therapy outcome. Nat Med 18: 1378–1385.

14. Alfonsi A, Cancès E, Turinici G, Ventura BD, Huisinga W (2005) Exact simulation of hybrid stochastic and deterministic models for biochemical systems. ESAIM Proc 14: 1–23.

15. Arora P, Dixit NM (2009) Timing the emergence of resistance to anti-HIV drugs with large genetic barriers. PLoS Computational Biology 5: e1000305.
